# Lewis Acidity and Basicity of Mixed Chlorometallate Ionic Liquids: Investigations from Surface Analysis and Fukui Function

**DOI:** 10.3390/molecules23102516

**Published:** 2018-09-30

**Authors:** Ying Liu, Juanfang Wang

**Affiliations:** College of Chemistry and Chemical Engineering, Inner Mongolia University, Hohhot 010021, China; 18647155024@163.com

**Keywords:** ionic liquid, Lewis acidity, surface analysis, Fukui function

## Abstract

Mixed chlorometallate ionic liquids (ILs) have been regarded as potential solvents, catalysts, and reagents for many organic processes. The acidity and basicity of these ILs were correlated with theoretically estimated parameters such as electrostatic surface potential maxima and minima, average local surface ionization energy, and Fukui and dual descriptor functions. The introduction of metal chloride into the anions would influence the acidity/basicity of ILs by withdrawing the electron density from the cationic counterpart. For the [C_4_mim]-based ILs with the mixed-metal anions, the acidity tends to attenuate while the basicity becomes stronger, as compared to the corresponding chloroaluminate ILs. However, the acidity of [(C_2_H_5_)_3_NH]-based ILs with the mixed-metal anions are greater than that of the net chloroaluminate ILs. The Fukui function values showed that most of the mixed chlorometallate ILs belong to bifunctional distribution. The mixed chlorometallate ILs both have electrophilic and nucleophilic sites, which would be beneficial for their applications.

## 1. Introduction

Room-temperature ionic liquids (ILs) have received much attention in the last few decades [1]. Ionic liquids exhibit interesting physical properties, such as low vapor pressure, a large liquidus range, high thermal stability, and favorable solvation behavior [2]. A key feature of ionic liquids is the large number of ion combinations that are possible, which suggests the feasibility of designing a suitable liquid for a specific task [3]. Due to the versatility in their physical and chemical properties, ionic liquids have been widely studied in many fields [4]. These fields usually include the production of biodiesel [5], the production of tensides/detergents by the Friedel-Crafts reaction [6,7,8], polymer chemistry [9], liquid-liquid extraction [10,11], and even enzymatic reactions [12]. On the other hand, although a great number of ionic liquids theoretically exist [13], only hundreds of them are commercially available and used as catalysts [14]. Among these ionic liquids, chlorometallate ionic liquids hold an important place in the catalysis field [15].

Primary applications of chloroaluminate ionic liquids are industrial Friedel-Crafts alkylation and acylation, and oligomerization and isomerization reactions of olefins and aromatics [16]. For example, a pilot-scale oligomerization of the olefins process at BP and an alkylation of the benzene process of Akzo-Nobel all used acidic chloroaluminate ionic liquids [(CH_3_)_3_NH]Cl-AlCl_3_ as catalysts [17]. Zhang et al. reported on the isomerization of *n*-pentane with [(C_2_H_5_)_3_NH]Cl-AlCl_3_ [18]. Huang et al. [19] and Wang et al. [20] studied the isomerization of dicyclopentadienes by [Hpy]Cl-AlCl_3_. In particular, the alkylation reaction of isobutane and butenes catalyzed by chloroaluminate ionic liquids, such as [C_4_mim]Cl-AlCl_3_ [21], [(C_2_H_5_)_3_NH]Cl-AlCl_3_ [22], and amide-AlCl_3_-based IL [23], have been studied intensively for years [15]. Compared with chloroaluminate ILs, the chlorometallate systems that only contain Fe-, Cu-, Zn-, In-, or Sn-based anions usually present lower Lewis acidity [24]. Thus, there are only comparatively few examples in their performance as acid catalysts. Cui et al. [25] have found that the alkylation of benzene with *p*-formaldehyde could be catalyzed by a series of acidic chlorometallate ionic liquids, such as [(C_2_H_5_)_3_NH]Cl-FeCl_3_, [(C_2_H_5_)_3_NH]Cl-ZnCl_2_, and [(C_2_H_5_)_3_NH]Cl-SnCl_2_. Gunaratne et al. also reported that phenols and catechols could be alkylated in weakly acidic chloroindate ionic liquids. Particularly, this acidic system could control the product selectivity and prevent polyalkylation that would occur using stronger acids [26]. In addition, [C_4_mim][FeCl_4_] has been used as a mild Lewis acid to catalyze the benzylation of arenes [27].

Adding metal salts to chlorometallate ionic liquids can either lead to changes in chlorometallate anion activity, or to the coordination of the added metal ions by chlorometallate anions [28]. This feature might be one of the key advantages for some reactions. For example, we used the [(C_2_H_5_)_3_NH]Cl-AlCl_3_-CuCl system for isobutane alkylation, and high quality gasoline was obtained [29,30]. Zinurov et al. [31] found that using the acidic [(CH_3_)_3_NH]Cl-AlCl_3_ ionic liquid doped with copper salts as catalysts could control the route of the isomerization of *n*-pentane. It is also found that adding titanium tetrachloride to the slightly acidic [C_4_mim]Cl-AlCl_3_ ionic liquid could lead to the formation of high yields of branched polymers [32]. Additionally, when the mixed chlorometallate [(C_2_H_5_)_3_NH]Cl-FeCl_3_-CuCl was used, the conversion and selectivity of the isobutene oligomerization could be enhanced significantly [33]. The best known example of catalysis in mixed chlorometallate ionic liquids may be the Institut Francais Du Petrole (IFP) Difasol process for olefin dimerization, which uses the [C_4_mim][AlCl_4_]-EtAlCl_2_-NiCl_2_ ionic liquid as a catalyst [34,35].

In order to improve the efficiency of IL-based processes, it is necessary to obtain accurate knowledge of physicochemical properties of ILs. Several experimental studies on the acidity of chlorometallate ILs have been reported. Using aromatic hydrocarbons as indicators, the acidity of HCl in chloroaluminate ILs can be determined by ultraviolet-visible (UV-vis) spectroscopy [36]. Osteryoung and co-workers [37,38], and Swadzźba-Kwasśny and co-workers [39] measured the Gutmann acceptor number (AN) of chlorometallate ILs using the chemical shift in the ^31^P NMR spectroscopy of triethylphosphine oxide (TEPO) as a probe molecule. X-ray photoelectron spectroscopy (XPS) was also employed to study the Lewis acidity and hydrogen bond basicity of halometallate-based ILs [40]. In addition, Kou [41] and Hu [42] used pyridine or acetonitrile as probe molecules to determine the acidity of chloroaluminate ILs by infrared spectroscopy.

The strength of interaction of a Lewis acid-base pair depends on the size, shape, and relative energies of the acid and the base. Consequently, the strength of a Lewis acid depends on the base that it is interacting with [15]. However, the impossibility of establishing a universal scale of Lewis acidity/basicity does not prevent the determination of the quantitative behavior of Lewis acids/bases [43]. There are a number of established Lewis acidity scales by experimental studies like the above mentioned, and the strength is also quantified by computational calculations [44]. Contreras et al. [45] have introduced two models of the Lewis molecular acidity: the excess electronic chemical potential and the local charge capacity. Furthermore, regional electrophilic and nucleophilic Fukui functions were also proposed to explain the Lewis acidity and basicity of ILs [46]. According to these models, several quantum-chemical parameters were used to determine the acid-base properties of the ILs. For example, Parveen et al. have performed a precise surface and Fukui analysis for several chlorometallate ionic liquids. The acidity and basicity of these ILs were correlated with theoretically parameters [47]. Similar works have also been done by Wu and co-workers [48].

As mentioned above, the isobutane-butene alkylation reaction catalyzed by mixed chlorometallate ILs is an important chemical process. The improvement of product selectivity is often attributed to the introduction of metal salts to the chloroaluminate ILs. Generally, it is believed that adding metal salts would reduce the super acidity of the chloroaluminate ILs [49]. However, the acidity of these ILs is still ambiguous. In addition, why are mixed chlorometallate ILs are better Lewis acidic catalysts than chloroaluminate ILs? What are the roles of anion and cation on the total acidity of ILs? These problems are also important for the better application of the mixed chlorometallate ILs. In this work, the acidity and basicity of mixed chlorometallate ILs were investigated by the electrostatic analysis of the molecular surface. In order to quantitatively determine the Lewis acidity of a region, the maximum surface electrostatic potential (*V*_s,max_) was employed. Additionally, the basicity of the ILs was estimated by the minimum surface electrostatic potential (*V*_s,min_) and the lowest average local ionization energy on the surface ($\bar{I}$_s,min_). Moreover, the Fukui function and the dual descriptor function were also applied to assess the roles of anions and cations on the Lewis acidity/basicity of ILs.

## 2. Results and Discussion

### 2.1. Acidity and Basicity of the Chlorometallate ILs

In this work, the most commonly used chloroaluminate ILs, i.e., [(C_2_H_5_)_3_NH]Cl-AlCl_3_ (triethylamine hydrochloride chloroaluminate IL), and [C_4_mim]Cl-AlCl_3_ (1-butyl-3-methylimidazolium chloroaluminate IL were selected for study. Some non-chloroaluminate ILs, [(C_2_H_5_)_3_NH]Cl-ZnCl_2_, and [C_4_mim]Cl-CuCl, were also employed for comparison. Figures S1–S16 shows the optimized geometries of these ILs. To some extent, the acidity of an IL depends on its ability to form electrostatic interactions with basic species. The surface electrostatic potential (*V*_s,max_) may be one useful tool for investigating and predicting the acidic region of ILs at the microscopic level. The larger magnitude of *V*_s,max_ usually indicates that the ions have a stronger interaction or acidity. Some quantum-chemical parameters are listed in Table 1.

For the same cation, the acidity of chlorometallate ILs (*V*_s,max_) showed a decrease in the order of Al > Zn > Cu, which is in agreement with the electronegativity trend of the three metals [37]. Different cations also significantly influence the Lewis acidity of the ILs with the same anion (e.g., [(C_2_H_5_)_3_NH]^+^[AlCl_4_]^−^ and [C_4_mim]^+^[AlCl_4_]^−^). When the [C_4_mim]^+^ cation was presented, the value of *V*_s,max_ could reach to 48.275 kcal/mol. Additionally, it is well known that the Lewis acidity of chloroaluminate ILs can be controlled by the mole fraction of aluminum chloride used for the IL preparation. If the mole ratio of aluminum chloride to organic salt is greater than 2:1, the chloroaluminate IL would exhibit super-acidity and [Al_2_Cl_7_]^−^ would be the dominated anion [50]. The surface analysis parameters support the above conclusion. The *V*_s,max_ value of [C_4_mim]^+^[Al_2_Cl_7_]^−^ is much higher than that of [C_4_mim]^+^[AlCl_4_]^−^: 52.596 vs. 48.275. Similarly, [(C_2_H_5_)_3_NH]^+^[Al_2_Cl_7_]^−^ has a larger *V*_s,max_ value than that of [(C_2_H_5_)_3_NH]^+^[AlCl_4_]^−^. The non-chloroaluminate ILs [(C_2_H_5_)_3_NH]^+^[ZnCl_3_]^−^ and [(C_2_H_5_)_3_NH]^+^[Zn_2_Cl_5_]^−^ have the same trend. Therefore, in accordance with the experimental data, the Lewis acidity of ILs will become stronger with the increase of the mole fraction of metal chloride.

**Figure 1:**
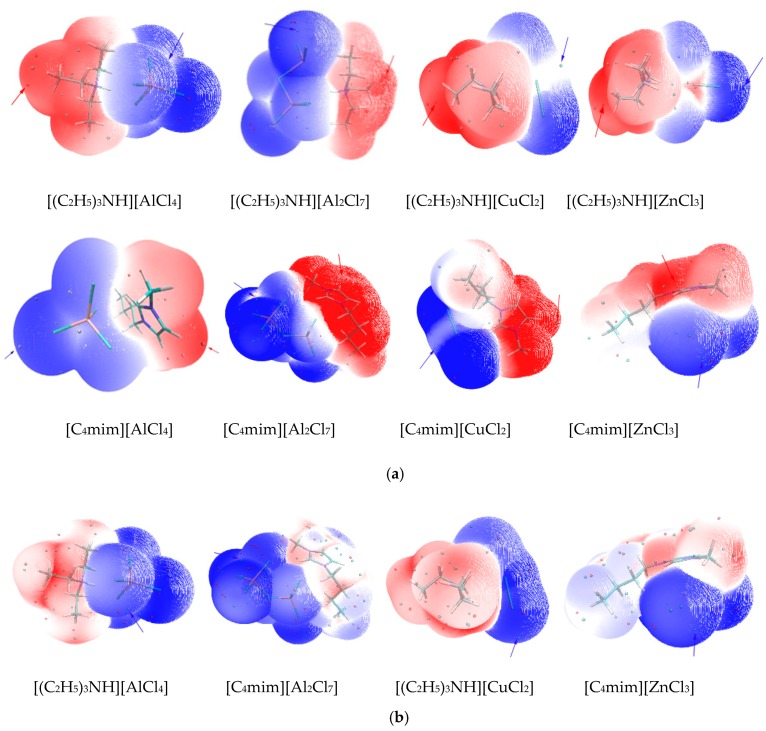
Molecular surface properties of chloroaluminate ILs: (**a**) the electrostatic potential with a 0.001 a.u. contour of the electron density and location of the *V*_s,max_ (red arrow) and *V*_s,min_ (blue arrow) on the ILs; (**b**) the average local ionization energy with a 0.001 a.u. contour of the electron density and $\bar{I}$_s,min_ on the ILs (blue arrow).

Figure 1 graphically illustrates the molecular surface properties of the ion pairs for the studied ILs. As Figure 1 shows, the locations of *V*_s,max_ for the ILs were all around the cations, which indicated that the acidity of the ILs resulted from the cation. This result was in conformity with the conclusions of other studies [4].

The magnitude of *V*_s,min_ can indicate the electrostatically reactive abilities of the molecules, while $\bar{I}$_s,min_ can show the electron-transfer ability. The two parameters often indicate the regional basicity of the ILs. Generally, a larger absolute value of *V*_s,min_ and a smaller $\bar{I}$_s,min_ usually indicate that the IL will exhibit a stronger basicity than the others. For the chlorometallate ILs, it is found that the sites of *V*_s,min_ and $\bar{I}$_s,min_ are all around the Cl atom in the anions (Figure 1), which means that the basicity of ILs is mainly focused on the anions. In general, the much larger size of the anions always leads to a more dispersed distribution of the negative charge. Therefore, the higher order complexation anions (e.g., [Al_2_Cl_7_]^−^ and [Zn_2_Cl_5_]^−^) would have less basicity, consistent with the results from the *V*_s,msx_ analysis. Additionally, the metal chloride could markedly affect the basicity of the chlorometallate ILs, as Table 1 shows.

### 2.2. Acidity and Basicity of the Mixed Chlorometallate ILs

Although chloroaluminate ILs have been widely used in solvent and catalysis processing, only a few surface analyses have focused on the mixed chlorometallate ILs (e.g., [(C_2_H_5_)_3_NH]Cl-AlCl_3_-CuCl, [C_4_mim]Cl-AlCl_3_-AgCl). Herein, a series of typical mixed chlorometallate ILs were chosen for investigation. These ILs are composed of the chloroaluminate anion and several other chlorometallate anions. The acidities of the ILs were compared with the conventional chloroaluminate ILs. Additionally, some ionic solvents for olefin absorption (e.g., benzene-CuAlCl_4_ and ether-CuClAl_4_ [51,52]) were also employed for comparison.

**Figure 2:**
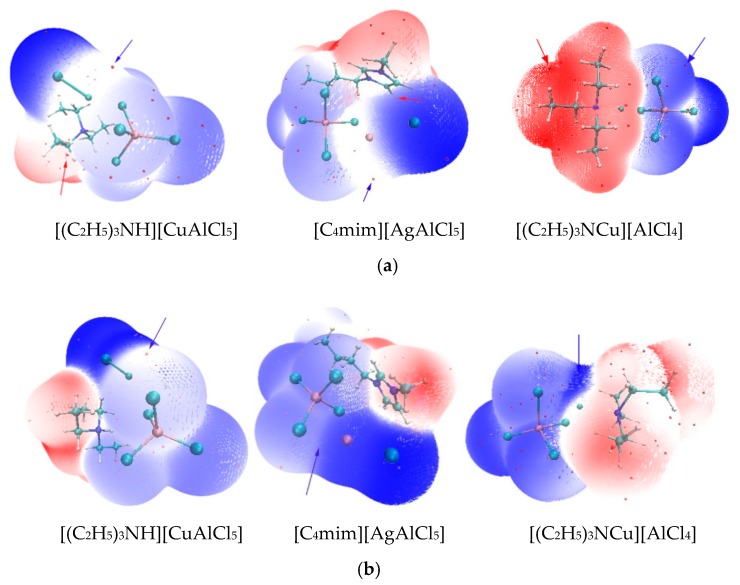
Molecular surface properties of mixed chlorometallate ILs: (**a**) electrostatic potential with a 0.001 a.u. contour of the electron density and the location of *V*_s,max_ (red arrow) and *V*_s,min_ (blue arrow) on the ILs; (**b**) average local ionization energy with a 0.001 a.u. contour of the electron density and $\bar{I}$_s,min_ on the ILs (blue arrow).

In general, the acidities of the chloroaluminate ILs were stronger than those of the corresponding mixed metal ILs; e.g., [C_4_mim]Cl-AlCl_3_ > [C_4_mim]Cl-AlCl_3_-CuCl (Table 1 and Table 2). Many studies believe that the Lewis acidity of chloroaluminate IL would be reduced by introducing another metal chloride [39,53]. Additionally, the ion pairs [C_4_mim]^+^[CuAlCl_5_]^−^ or [C_4_mimCu]^+^[AlCl_4_]^−^ could be formed in [C_4_mim]Cl-AlCl_3_-CuCl. As Table 2 shown, a surface analysis found that [C_4_mim]^+^[CuAlCl_5_]^−^ and [C_4_mimCu]^+^[AlCl_4_]^−^ all led to smaller *V*_s,max_ values. The location of *V*_s,max_ in [C_4_mim]^+^[AlCl_4_]^−^ was around the H atom of an imidazolium ring, similar to the *V*_s,max_ sites in copper−containing ILs (Figure 1 and Figure 2). Owing to the smaller values of *V*_s,max_, copper-containing IL [C_4_mim]Cl-AlCl_3_-CuCl exhibited weaker acidity than that of [C_4_mim]Cl-AlCl_3_. These results are consistent with the previous conclusions.

From the determination of the Gutmann acceptor number, we learned that the acidity of [(C_2_H_5_)_3_NH]Cl-AlCl_3_-CuCl is larger than that of [(C_2_H_5_)_3_NH]Cl-AlCl_3_ [54]. Some researchers have considered that [(C_2_H_5_)_3_NCu]^+^ and [AlCl_4_]^−^ are the dominant ions of [(C_2_H_5_)_3_NH]Cl-AlCl_3_-CuCl. In other words, CuCl coordinates with the [(C_2_H_5_)_3_NH]^+^ cation to form [(C_2_H_5_)_3_NCu]^+^ and to release HCl [55]. However, the *V*_s,max_ value of [(C_2_H_5_)_3_NCu]^+^[AlCl_4_]^–^ is much smaller than that of [(C_2_H_5_)_3_NH]^+^[AlCl_4_]^−^ (Table 1), which is not consistent with the experimental results. By contrast, the *V*_s,max_ of [(C_2_H_5_)_3_NH]^+^[CuAlCl_5_]^−^ is larger than that of the corresponding net chloroaluminate IL, indicating that [(C_2_H_5_)_3_NH]^+^[CuAlCl_5_]^−^ exhibits a stronger acidity than that of [(C_2_H_5_)_3_NH]^+^[AlCl_4_]^−^. For comparison, the benzene–CuAlCl_4_ solvent was also employed for the surface analysis, and the *V*_s,max_ of benzene-CuAlCl_4_ was higher than that of benzene-[AlCl_4_]^−^. All of the results suggest that a large number of [(C_2_H_5_)_3_NH]^+^ and [CuAlCl_5_]^−^ should be presented in the ionic liquid [(C_2_H_5_)_3_NH]Cl-AlCl_3_-CuCl.

*V*_s,min_ and $\bar{I}$_s,min_ can determine the ability of ILs to act as H-bond acceptors. No matter which type of cation is, the sites of *V*_s,min_ for the studied ILs are all around the Cl atom of the anion. Nonetheless, the *V*_s,min_ of the chlorometallate ILs was more negative than those of the net chloroaluminate ILs. Therefore, the mixed chlorometallate ILs would be stronger H-bond acceptors with respect to the conventional chloroaluminate ILs.

As Figure 1 and Figure 2 shown, the $\bar{I}$_s,min_ site of the chloroaluminate ILs were located around the chloride atom, whereas the $\bar{I}$_s,min_ locations of the mixed chlorometallate ILs were all around the transition metal atoms (e.g., Cu, Ag) of the ions. A low value of $\bar{I}$_s_ suggests that the electron at this position was not tightly bounded, and the site with the lowest $\bar{I}$_s_ (i.e., $\bar{I}$_s,min_) on the surface is usually recognized as the location where an electron is mostly inclined to escape from the molecule. Therefore, the electron of the transition metal atom may leave the molecular surface more easily, leading to $\bar{I}$_s,min_ sites around the transition metal atom in mixed chlorometallate ILs. In addition, for triethylamine hydrochloride-based ILs, less negative interaction energy of [(C_2_H_5_)_3_NCu]^+^[AlCl_4_]^−^ (Table 2) indicates that the cation–anion interaction of [(C_2_H_5_)_3_NCu]^+^[AlCl_4_]^−^ is weaker than that of [(C_2_H_5_)_3_NH]^+^[CuAlCl_5_]^−^. Thus, the [(C_2_H_5_)_3_NCu]^+^ cations are more inclined to leave the [AlCl_4_]^−^ anions and become unstable.

**Figure 3:**
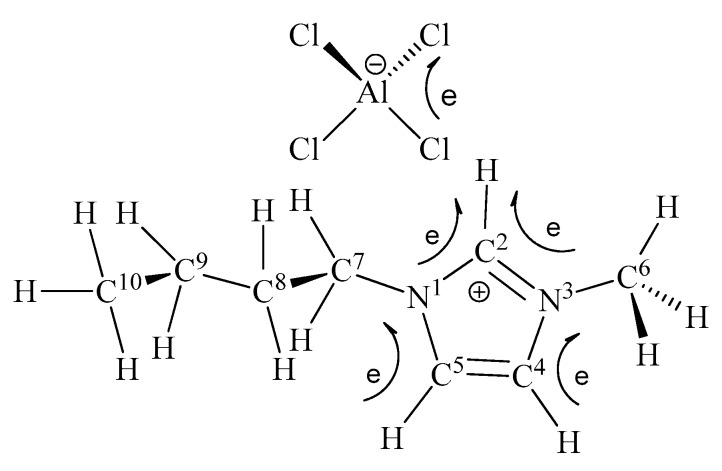
Direction of the charge transfer when AlCl_3_ approaches the ion pair unit (C_4_mim-Cl).

Generally, the *V*_s,max_ values have a strong dependence on the cation-anion charge transfer (*Q*_CT_). To further investigate the Lewis acidity, the atomic charges and charge transfer properties of the ILs under study were calculated, and the data are listed in Table 3. Generally, chlorometallate-based ILs were synthesized by direct addition of the required metal chloride MCl*_x_* to C_4_mim-Cl in an appropriate molar ratio and under an inert atmosphere. Thus, the charge transfer *Q*_CT_ should take place from the ion pair unit C_4_mim-Cl, in which the Cl atom of C_4_mim-Cl is close to the imidazole ring, and it forms a H-bond with the C^2^-H moiety, to the MCl*_x_* unit. Thus, the charge transfer in the IL can be described as *Q*_CT_(IP→MCl*_x_*). As Figure 3 and the data collected in Table 3 show, the charge transfer *Q*_CT_ of [C_4_mim]^+^[AlCl_4_]^−^ indeed occurs from the ion pair unit C_4_mim-Cl to the AlCl_3_ unit. Moreover, a larger amount of charge transfer corresponds to the stronger acidity of the ILs. Relative to [C_4_mim]^+^[CuAlCl_5_]^−^, *Q*_CT_(IP→MCl*_x_*) appears to be greater in magnitude for the [C_4_mim]^+^[AlCl_4_]^−^ that exhibits stronger acidity: 407 *m*_e_ vs. 345 *m*_e_. Similarly, [C_4_mim]^+^[Zn_2_Cl_5_]^−^ shows stronger acidity than that of [C_4_mim]^+^[ZnCl_3_]^−^. These results are consistent with the conclusions drawn from the *V*_s,max_ values.

### 2.3. Analysis of Fukui Function for Mixed Chlorometallate ILs

The Fukui function (FF) is an important concept in conceptual density functional theory, which is defined as Equation (1) [56]:

(1) $$f\left(r\right)={\left(\frac{\partial \rho (r)}{\partial N}\right)}^{+}{=\rho }_{N+1}\left(r\right)-\rho_{N}\left(r\right)$$

where *N* is the number of electrons in the present system. Cerda-Monje et al. have proposed that the condensed to atom electrophilic FF $f_{\mathrm{atom}}^{+}$ can be considered as the natural distributor of Lewis molecular acidity (Equation (2)), and the nucleophilic FF $f_{\mathrm{atom}}^{-}$ is the corresponding distributor of Lewis molecular basicity (Equation (3)) [45,46]. Thus, the condensed Fukui function of the molecule might be readily calculated by using the charges of the atoms:

(2) $$f_{\mathrm{atom}}^{+}=\rho_{N+1}\left(r\right)-\rho_{N}\left(r\right)=q_{N}^{\mathrm{atom}}-q_{N+1}^{\mathrm{atom}}$$

(3) $$f_{\mathrm{atom}}^{-}=\rho_{N}\left(r\right)-\rho_{N-1}\left(r\right)=q_{N-1}^{\mathrm{atom}}-q_{N}^{\mathrm{atom}}$$

If we integrate over the corresponding basins $\Omega_{k}^{\pm }$ (local area of a molecule), the basins FF could be produced as Equation (4):

(4) $$N_{\Omega }^{\pm }=\int_{\Omega_{\mathrm{atom}}^{\pm }}\rho \left(r\right)dr=\underset{\mathrm{atom}\in \Omega_{\mathrm{atom}}^{\pm }}{\sum }f_{\mathrm{atom}}^{\pm }$$

The basins $\Omega_{\mathrm{atom}}^{\pm }$ represent the maximum molecular regions of the electrophilic FF ($\Omega_{\mathrm{atom}}^{+}$) and nucleophilic FF ($\Omega_{\mathrm{atom}}^{-}$), respectively. Thus, the region with the larger $N_{\Omega }^{+}$ is the favorable site to accept the electron charge, while the region with the greater $N_{\Omega }^{-}$ is the location that prefers to donate the electron charge. According to the suggestion of Cerda-Monje et al., the concept of “normal distribution’’, ‘‘bifunctional distribution’’, and ‘‘border-line distribution’’ was employed to describe the studied ILs [46]. The “normal distribution” indicates that the Lewis molecular acidity ($N_{\Omega }^{+}$) is mainly centered on the cation fragment, and the Lewis molecular basicity ($N_{\Omega }^{-}$) is mostly centered on the anion. In our cases, the $N_{\Omega }^{+}$ of the cation was usually greater than 0.9 eV, and $N_{\Omega }^{-}$ was less than 0.2 eV. Meanwhile, the values of the anion moiety should be $N_{\Omega }^{+}$ < 0.2 eV and $N_{\Omega }^{-}$ > 0.9 eV, respectively. The “bifunctional distribution” usually indicates that both the electrophilic and nucleophilic reactive sites are located at the same region of the ILs. When $N_{\Omega }^{+}$ and $N_{\Omega }^{-}$ are great than 0.5 eV and 0.8 eV, respectively, the anion will exhibit “bifunctional distribution”. For the cation, the “bifunctional distribution” means that $N_{\Omega }^{+}$ > 0.8 eV and $N_{\Omega }^{-}$ > 0.5 eV. The remaining values belong to the “borderline distribution”, which will not clearly indicate the location that prefers to accept or donate the electron charge.

**Figure 4:**
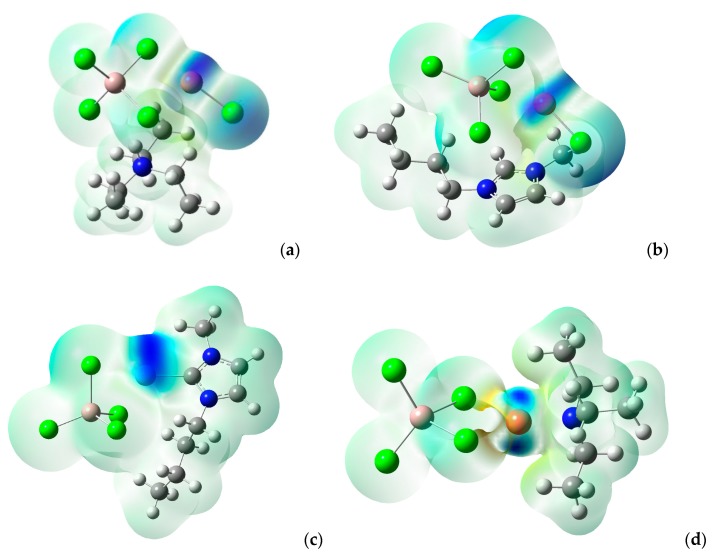
Electron donor Fukui function $f_{\mathrm{atom}}^{-}$ electron-density isosurface plot at 0.004 a.u: (**a**) [(C_2_H_5_)_3_NH]^+^[CuAlCl_5_]^−^; (**b**) [C_4_mim]^+^[CuAlCl_5_]^−^; (**c**) [C_4_mimAg]^+^[AlCl_4_]^−^; (**d**) [(C_2_H_5_)_3_NCu]^+^[AlCl_4_]^−^; the blue color around Cu or Ag indicates that the transition metal atom is a favorite site for accepting the electrophilic reagent.

As the data collected in Table 4 shown, most net chlorometallate ILs (e.g., [(C_2_H_5_)_3_NH]^+^[AlCl_4_]^−^, [(C_2_H_5_)_3_NH]^+^[ZnCl_3_]^−^) exhibited a normal distribution, indicating that the Lewis acidity and basicity were located on the cation and the anion, respectively. However, the IL [(C_2_H_5_)_3_NH]^+^[CuAlCl_5_]^−^ showed a bifunctional distribution, implying that the location to accept and to donate the electronic charges were all situated on the anions [CuAlCl_5_]^−^. As Figure 4 shows, the presence of the Cu atom enhances the ability of electrophilic attack of the anion. In previous work, we found that the [(C_2_H_5_)_3_NH]^+^[CuAlCl_5_]^−^ ionic liquid could produce better alkylation gasoline than that of net [(C_2_H_5_)_3_NH]^+^[AlCl_4_]^−^ [29,30]. According to the experimental results, the authors proposed a possible mechanism and raised a reasonable explanation for the possible mechanism, that is, the higher catalytic selectivity is attributed to the interaction between *i*C_4_^+^ carbonium ion and the transition metal atom [57]. As demonstrated in this work, the IL [(C_2_H_5_)_3_NH]^+^[CuAlCl_5_]^−^ exhibited a bifunctional distribution ($N_{\Omega }^{+}$ > 0.5 eV and $N_{\Omega }^{-}$ > 0.8 eV for the anion), indicating that the anion not only can provide Lewis acidity, but it can also absorb a considerable number of *i*C_4_^+^ carbonium ion.

A dual descriptor is another useful function that is used to reveal the reactive sites [58]. Based on the Fukui function, the dual descriptor can be easily calculated by $f_{\mathrm{atom}}^{+}$ and $f_{\mathrm{atom}}^{-}$ (Equation (5)).

(5) $$∆f_{\mathrm{atom}}=f_{\mathrm{atom}}^{+}-f_{\mathrm{atom}}^{-}$$

If the distribution of $∆f_{\mathrm{atom}}$ around a site *A* is more positive than another site *B*, then one can say that *A* is a more favorable site for nucleophilic attack than *B*, and in the meantime, *B* is a more preferential site for electrophilic attack than *A*. As can be seen from Table S2, the Cu atom of [(C_2_H_5_)_3_NH]^+^[CuAlCl_5_]^−^ gave a more negative $∆f_{\mathrm{atom}}$ value than that of any other atom; meanwhile, the Al atom showed a maximum $∆f_{\mathrm{atom}}$ value. These results again indicate that the mixed chlorometallate anion [CuAlCl_5_]^−^ had both favorable sites for nucleophilic and electrophilic properties with respect to the net chloroaluminate anion [AlCl_4_]^−^. The ionic liquid [(C_2_H_5_)_3_NH]^+^[AgAlCl_5_]^−^ also exhibited bifunctional distribution. The anion $N_{\Omega }^{\pm }$ values were 0.879 and 0.932, respectively.

Fukui analysis further indicates that many mixed chlorometallate ILs, such as [C_4_mim]Cl-AlCl_3_-CuCl, [C_4_mim]Cl-AlCl_3_-AgCl, [(C_2_H_5_)_3_NH]Cl-AlCl_3_-ZnCl_2_, benzene-CuAlCl_4_, and ether-AgAlCl_4_ all can show bifunctional behavior. For imidazolium-based ionic liquids, the data of Fukui function shows that $N_{\Omega }^{+}$ > 0.5 eV and $N_{\Omega }^{-}$ > 0.8 eV for [CuAlCl_5_]^−^ anion, and $N_{\Omega }^{+}$ > 0.8 eV and $N_{\Omega }^{-}$ > 0.5 eV for [C_4_mimAg]^+^ cation. The results strongly implies that both nucleophilic and electrophilic sites are located on the same ion of [C_4_mim]Cl-AlCl_3_-MCl (M=Cu, Ag) ILs. Additionally, ∆*f* of the transition metal atoms in [(C_2_H_5_)_3_NH]Cl-AlCl_3_-CuCl, [C_4_mim]Cl-AlCl_3_-CuCl, [(C_2_H_5_)_3_NH]Cl-AlCl_3_-AgCl, ether-AgAlCl_4_, and [(C_2_H_5_)_3_NH]Cl-AlCl_3_-ZnCl_2_ ILs all gave the most negative values (see Tables S2, S3, S8, S9 and S12)), suggesting that the corresponding atoms were the most favorable sites for electrophilic (*i*C_4_^+^) attack. The transition metal mixed ILs could provide a higher catalytic selectivity of C_4_ alkylation as compared to the traditional catalysts (e.g., H_2_SO_4_) and the conventional chlorometallate ILs (e.g., [C_4_mim]Cl-AlCl_3_) [57]. Other than as a Lewis acid, it is believed that the mixed chlorometallate ILs would attract more *i*C_4_^+^ to the surrounding transition metal atoms. Thus, better catalytic selectivity can be obtained due to an increase of the I/O (*i*C_4_^+^ to olefins) ratio around the anion.

## 3. Methods

Recently, an assessment of density functional theory and Møller-Plesset perturbation theory for ILs has been performed [59]. Li et al. have recommended that the density functional of the Minnesota family of the M0X type with a diffusion function basis set (aug-cc-pvdz) would give reliable results for IL calculations [60]. Similarly, Zahn et al. noted that dispersion-corrected density functionals, such as B3LYP/6-31++G**-gd3bj (DFT-D3), could also lead to reliable results for the IL calculations [61]. In this work, we selected the M06-2X/aug-cc-pVDZ level of theory to optimize the geometries of the ILs under study [62]. This level is known to afford good geometry at low computational cost, which is particularly appropriate to be used for the calculation of cation-anion interactions [63]. To calculate the Ag-containing ILs, the basis set aug-cc-pVDZ-PP (Ag, 28 core electrons) that was obtained from the EMSL Basis Set Exchange was employed, and the Dunning’s correlation-consistent basis set aug-cc-pVDZ was applied for the remaining atoms [64]. Optimized geometries of the studied ILs were confirmed as minima on the potential energy surface by the absence of imaginary vibrational frequencies. The Gaussian 09 package [65] was employed to carry out all of these computations.

In this work, the scheme of restrained electrostatic potentials (RESP) employing the ChelpG point selection algorithms and the density matrix partitioning scheme Natural Population Analysis (NPA) were employed to calculate the atomic partial charges in the ionic liquids. Rigby and Izgoridina [66] have recently assessed different atomic partial charge schemes for polarization and charge transfer effects in ionic liquids. They suggest that RESP schemes are preferred when producing atomic partial charges, as RESP can show an appreciable degree of charge transfer. Among the RESP schemes, ChelpG is the least systematic for ion pairs of ionic liquids, and it should be used. Therefore, the RESP-ChelpG model was selected, and the atomic charges were finally calculated by the Multiwfn 3.6 (dev) program [67]. Because of largely basis set independent, the NPA scheme was also selected for the calculation. NPA atomic charges were obtained by the natural bond orbital (NBO) program [68] using M06-2X/aug-cc-pVDZ(-PP) for the ion pairs. Though the charges from the two schemes were slightly different in magnitude, the conclusions drawn are almost no difference.

Electrostatic potential, *V*(***r***), has been widely used for prediction of nucleophilic and electrophilic sites, as well as the molecular recognition mode, which is defined as Equation (6):

(6) $$V\left(r\right)=\underset{A}{\sum }\frac{Z_{A}}{\left|{r-R}_{A}\right|}-\int \frac{{\rho (r}^{\prime })}{\left|r-r^{\prime }\right|}dr^{\prime }$$

where ***R****_A_* and *Z_A_* denote the position vector and the nuclear charge of atom *A*, respectively. If the pseudo-potential was used, *Z_A_* would be the number of explicitly expressed electrons. The analyses of electrostatic potential are commonly performed on molecular Van der Waals (vdW) surfaces. In our analyses, we took the 0.001 a.u. isosurface of the electron density as the vdW surface. Although the definition of such a surface is arbitrary, the above definition may well reflect specific electron structural features of a molecule. The strength and orientation of weak interactions, including regional acidity, hydrogen bonding, and halogen bonding, can be well predicted and explained by analysing the magnitudes and positions of minima (*V*_s,min_) and maxima (*V*_s,max_) on the surface [67]. The surface average local ionization energy $\bar{I}\left(r\right)$ can be written as Equation (7):

(7) $$\bar{I}\left(r\right)=\underset{i}{\sum }\frac{\rho_{i}\left(r\right)\left|\varepsilon_{i}\right|}{\rho \left(r\right)}$$

where *ρ*(***r***) and *ε_i_* are the electron density function and orbital energy of the *i*th molecular orbital, respectively. Typical DFT functionals are suitable for computing $\bar{I}\left(r\right)$. A lower value of $\bar{I}\left(r\right)$ indicates that the electrons at this point are more weakly bounded. It is proven that the minima of the average local ionization energy ($\bar{I}$_s,min_) on the vdW surface are good indicators to reveal the basicity of ILs [69].

The Multiwfn 3.6 program was employed to determine the values of *V*_s,min_, *V*_s,max_, and $\bar{I}$_s,min_, respectively. Using the optimized geometries, wave functions, and electrostatic potential cube files were generated from Gaussian 09. The graphs of the electrostatic potential and the average local ionization energy were depicted by the visualization tool, VMD [70]. The regional Fukui function and dual descriptor, which are two well-known local descriptors for electron gain and donation, were also calculated by the Multiwfn 3.6 (dev) program.

Cation–anion interaction energies of the ILs were calculated according to Equation (8). Furthermore, the basis set superposition error (BSSE) was corrected using the counterpoise method when calculating the interaction energies [71]:

∆*E*_IL_(kcal/mol) = 627.51 × [*E*_ion-pair_ (au) − *E*_cation_ (au) − *E*_anion_ (au)] (8)

## 4. Conclusions

Using a surface analysis, Fukui function, and a dual descriptor function, the acidity and basicity of the mixed chlorometallate ILs were investigated. The quantum-chemical parameter *V*_s,max_ was adopted to quantitatively describe the Lewis acidity of ILs, while *V*_s,min_, and $\bar{I}$_s,min_ were proposed to interpret their basicity. Many mixed metal-containing ILs exhibit weaker Lewis acidity with respect to the corresponding chloroaluminate ILs. However, due to the presence of the coordinated anions MAlCl_5_^−^ (M=Cu, Ag), [(C_2_H_5_)_3_NH]Cl-AlCl_3_-MCl has higher acid strength than that of [(C_2_H_5_)_3_NH]Cl-AlCl_3_. Mixed chlorometallate ILs have more complicated ion species, while the MAlCl_5_^−^ ion and MAlCl_4_ complex would abound in the ILs. The analyses of the Fukui function and the dual descriptor function show that most of the mixed chlorometallate ILs belong to the bifunctional distribution. Both the electrophilic and the nucleophilic sites are located in the same region of ILs, which have benefits in improving the product selectivity of the isobutane–butene alkylation process. These results can provide a deep understanding of the nature of the chlorometallate ILs. Moreover, they might give useful instruction for preparing novel functional ILs.

## Figures and Tables

**Table 1:** Cation-anion interaction energies and surface parameters of the Room-temperature ionic liquids (ILs) (kcal/mol).

[Cation][Anion]	*V*_s,max_	*V*_s,min_	$\bar{I}$_s,min_^1^	∆*E*
[(C_2_H_5_)_3_NH][AlCl_4_]	42.430	−33.706	10.224	−90.361
[(C_2_H_5_)_3_NH][Al_2_Cl_7_]	52.596	−30.145	10.480	−96.637
[(C_2_H_5_)_3_NH][CuCl_2_]	36.104	−41.120	8.730	−96.637
[(C_2_H_5_)_3_NH][ZnCl_3_]	41.400	−39.743	9.423	−92.871
[C_4_mim][Cl]	40.53	−59.29	5.81	−90.34
[C_4_mim][AlCl_4_]	48.275	−36.010	10.048	−84.086
[C_4_mim][Al_2_Cl_7_]	52.596	−30.145	10.480	−77.184
[C_4_mim][CuCl_2_]	42.003	−45.617	8.337	−84.086
[C_4_mim][Cu_2_Cl_3_]	43.950	−40.194	9.324	−56.476
[C_4_mim][ZnCl_3_]	42.585	−33.918	9.710	−85.969
[C_4_mim][Zn_2_Cl_5_]	44.895	−37.392	9.834	−75.765

^1^ The unit of $\bar{I}$_s,min_ is eV.

**Table 2:** Cation-anion interaction energies and the surface parameters of the ILs (kcal/mol).

[Cation][Anion]	*V*_s,max_	*V*_s,min_	$\bar{I}$_s,min_^1^	∆*E*
[(C_2_H_5_)_3_NH][CuAlCl_5_]	43.261	−37.11	9.261	−88.479
[(C_2_H_5_)_3_NH][AgAlCl_5_]	43.201	−37.336	9.243	−86.854
[(C_2_H_5_)_3_NCu][AlCl_4_]	27.965	−23.682	10.330	−82.992
[(C_2_H_5_)_3_NCu][Al_2_Cl_7_]	28.230	−20.229	9.286	−81.109
[(C_2_H_5_)_3_NAg][Al_2_Cl_7_]	26.725	−15.995	9.692	−80.579
[(C_2_H_5_)_3_NH][ZnAlCl_6_]	45.011	−35.572	9.677	−85.969
[C_4_mim][CuAlCl_5_]	43.445	−36.603	9.218	−84.086
[C_4_mim][AgAlCl_5_]	45.866	−37.470	9.257	−83.952
[benzene][CuAlCl_4_]	35.085	−20.596	10.891	−26.982
[benzene][AlCl_4_]^−^	33.680	−28.760	9.724	−28.987
[ether][CuAlCl_4_]	35.305	−23.540	10.871	−15.060
[ether][AlCl_4_]^−^	40.184	−24.125	7.182	−7.530

^1^ The unit of $\bar{I}$_s,min_ is eV.

**Table 3:** The magnitude of the charge transfer from the unit (C_4_mim-Cl) to the metal chloride [*Q*_CT_(IP→MCl*_x_*)], and the restrained electrostatic potentials (RESP) charges for the ILs under study ^1^.

[Cation][Anion]	*Q*_CT_	N1	C2	H(C2)	N3	C4	H(C4)	C5
[C_4_mim][Cl]	−	−0.045	0.008	0.273	0.043	−0.142	0.225	−0.226
[C_4_mim][AlCl_4_]	−0.407(−0.419)	0.136	−0.127	0.239	0.165	−0.223	0.261	−0.219
[C_4_mim][CuAlCl_5_]	−0.345(−0.378)	0.171	−0.073	0.244	0.149	−0.248	0.305	−0.256
[C_4_mim][ZnCl_3_]	−0.297(−0.267)	0.093	−0.115	0.234	0.098	−0.078	0.253	−0.187
[C_4_mim][Zn_2_Cl_5_]	−0.334(−0.368)	0.087	−0.114	0.211	0.123	−0.095	0.256	−0.198

^1^ All values are given in a.u. The atomic labels are shown in Figure 3. The values in parentheses are the amount of charge transfer computed with the Natural Population Analysis (NPA) scheme.

**Table 4:** Electrophilic ($N_{\Omega }^{+}$ ) and nucleophilic ($N_{\Omega }^{-}$ ) Fukui function values over the cations and anions for the studied ILs (eV).

[Cation][Anion]	Cation		Anion		Min. ∆*f*_atom_	Max. ∆*f*_atom_	Acidity/Basicity Distribution
	$N_{\Omega }^{+}$	$N_{\Omega }^{-}$	$N_{\Omega }^{+}$	$N_{\Omega }^{-}$			
[(C_2_H_5_)_3_NH][AlCl_4_]	0.930	0.081	0.063	0.918	Cl	H	Normal
[(C_2_H_5_)_3_NH][ZnCl_3_]	0.917	0.052	0.948	0.065	Cl	H	Normal
[(C_2_H_5_)_3_NH][CuAlCl_5_]	0.271	0.081	0.702	0.918	Cu	Al	Bifunctional
[(C_2_H_5_)_3_NCu][AlCl_4_]	0.862	0.815	0.131	0.185	Cu	Al	Bifunctional
[(C_2_H_5_)_3_NH][AgAlCl_5_]	0.075	0.068	0.879	0.932	Ag	Cl	Bifunctional
[C_4_mim][AlCl_4_]	0.914	0.084	0.083	0.916	Cl	C	Normal
[C_4_mim][CuCl_2_]	0.925	0.073	0.092	0.908	Cu	C	Normal
[C_4_mim][CuAlCl_5_]	0.273	0.079	0.719	0.921	Cu	Al	Bifunctional
[C_4_mimAg][AlCl_4_]	0.816	0.804	0.117	0.196	Ag	H	Bifunctional
[(C_2_H_5_)_3_NH][ZnAlCl_6_]	0.678	0.057	0.313	0.943	Zn	Al	Bifunctional ^1^
[Benzene][CuAlCl_4_]	0.166	0.109	0.913	0.924	Cl	Cu	Bifunctional
[Ether][CuAlCl_4_]	0.423	0.156	0.575	0.844	Cu	Al	Bifunctional

^1^ [(C_2_H_5_)_3_NH][ZnAlCl_6_] is only close to the standard of bifunctional distribution for anion: $N_{\Omega }^{+}$ > 0.5 eV and $N_{\Omega }^{-}$ > 0.8 eV.

## Supplementary Materials

The following are available online, Figures S1–S16: structures of optimized ionic liquids, Tables S1–S12: calculation data of Fukui function and dual description, Tables S13–S28: Cartesian coordinates of ILs.
