# Supplementary material for: Lewis Acidity and Basicity of Mixed Chlorometallate Ionic Liquids: Investigations from Surface Analysis and Fukui Function
Source: Molecules. 2018 Sep 30;23(10):2516. doi: 10.3390/molecules23102516 (PMC6222785; doi:10.3390/molecules23102516)
Supplement: Supplementary file 1 [file molecules-23-02516-s001.pdf]

Lewis acidity and basicity of mixed chlorometallate ionic liquids: investigations from surface analysis and Fukui function

Ying Liu * and Juanfang Wang

College of Chemistry and Chemical Engineering, Inner Mongolia University, Hohhot, 010021, China;

* Correspondence: celiuy@imu.edu.cn; Tel.: +86-471-4992981

Supplementary Materials

List of contents:

- Structure of ILs with carbon atoms numbered page S2

- Calculation data of Fukui function and dual description page S3–S11

- Cartesian coordinates of optimized ionic liquids page S12–S26

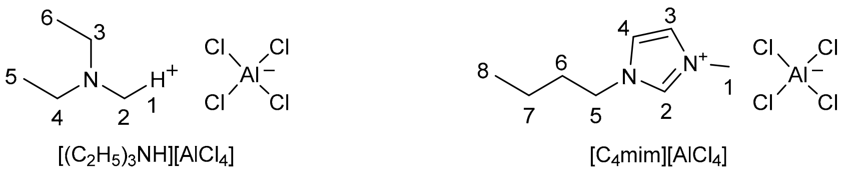

Scheme 1. Structure of $[(C_2H_5)_3NH][AlCl_4]$ and $[C_4mim][AlCl_4]$ with carbon atoms numbered.

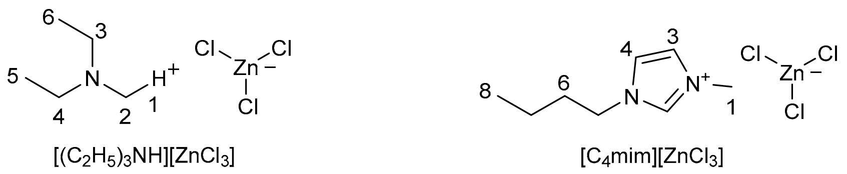

Scheme 2. Structure of $[(C_2H_5)_3NH][ZnCl_3]$ and $[C_4mim][ZnCl_3]$ with carbon atoms numbered.

Table S1. Electrophilic (N_{Ω}^{+}) and nucleophilic (N_{Ω}^{-}) Fukui function and dual description values for the IL $[(C_2H_5)_3NH][AlCl_4]$ (eV)

Atom	q_{N-1}	f^-	q_N	q_{N+1}	f^+	Δf
C	-0.0716	0.0057	-0.0773	-0.1534	0.0762	0.0705
C	0.0222	0.0019	0.0203	0.0030	0.0173	0.0154
N	0.0695	-0.0027	0.0722	0.0720	0.0002	0.0029
H	0.0662	0.0109	0.0553	-0.0545	0.1099	0.0990
H	0.0445	0.0047	0.0398	-0.0600	0.0998	0.0952
H	0.0503	0.0008	0.0496	0.0248	0.0248	0.0240
H	0.0555	0.0040	0.0514	0.0169	0.0345	0.0305
Al	0.4124	0.0515	0.3609	0.3483	0.0126	-0.0389
Cl	-0.0767	0.1825	-0.2592	-0.2660	0.0069	-0.1756
Cl	-0.1882	0.0655	-0.2537	-0.2590	0.0053	-0.0601
Cl	0.0824	0.3374	-0.2550	-0.2803	0.0253	-0.3122
Cl	0.0199	0.2816	-0.2617	-0.2742	0.0125	-0.2691
H	0.0469	0.0074	0.0395	-0.0716	0.1111	0.1037
C	-0.0789	0.0032	-0.0822	-0.0989	0.0168	0.0136
H	0.0508	0.0168	0.0340	0.0012	0.0328	0.0160
H	0.0597	0.0053	0.0544	0.0305	0.0238	0.0185
H	0.0259	-0.0058	0.0317	0.0197	0.0120	0.0178
C	-0.0737	0.0040	-0.0777	-0.1010	0.0234	0.0194
H	0.0369	-0.0052	0.0421	0.0256	0.0165	0.0217
H	0.0652	0.0097	0.0554	0.0178	0.0376	0.0279
H	0.0483	0.0117	0.0366	-0.0064	0.0430	0.0313
C	0.0219	-0.0002	0.0221	0.0059	0.0162	0.0164
H	0.0354	-0.0092	0.0446	0.0282	0.0164	0.0256
H	0.0591	0.0138	0.0453	-0.0265	0.0718	0.0579
C	0.0212	0.0023	0.0189	-0.0052	0.0241	0.0217
H	0.0563	0.0123	0.0440	-0.0455	0.0895	0.0772
H	0.0442	-0.0022	0.0465	0.0195	0.0270	0.0292
H	0.0946	-0.0076	0.1023	0.0965	0.0058	0.0134
Cation	N_{Ω}^{-}	0.0814		N_{Ω}^{+}	0.9304	
Anion	N_{Ω}^{-}	0.9184		N_{Ω}^{+}	0.0626	

Table S2. Electrophilic (N_{Ω}^{+}) and nucleophilic (N_{Ω}^{-}) Fukui function and dual description values for the IL $[(C_2H_5)_3NH][CuAlCl_5]$ (eV)

Atom	q_{N-1}	f^-	q_N	q_{N+1}	f^+	Δf
C	-0.0724	0.0048	-0.0773	-0.0852	0.0079	0.0031
C	0.0122	-0.0007	0.0129	0.0103	0.0026	0.0034
N	0.0712	-0.0029	0.0740	0.0745	-0.0005	0.0024
H	0.0648	0.0050	0.0598	0.0460	0.0139	0.0089
H	0.0454	0.0045	0.0408	0.0313	0.0095	0.0050
H	0.0148	-0.0138	0.0287	0.0285	0.0002	0.0140
H	0.0487	0.0037	0.0450	0.0380	0.0070	0.0033
Al	0.3878	0.0081	0.3798	0.2290	0.1508	0.1427
Cl	-0.1973	0.0336	-0.2308	-0.3315	0.1007	0.0671
Cl	-0.1716	0.0285	-0.2002	-0.2612	0.0610	0.0325
Cl	-0.1795	0.0515	-0.2311	-0.3182	0.0872	0.0356
Cl	-0.0858	0.0720	-0.1579	-0.2483	0.0904	0.0183

H	0.0487	0.0145	0.0342	0.0229	0.0113	-0.0032
C	-0.0765	0.0035	-0.0799	-0.0919	0.0119	0.0085
H	0.0297	-0.0049	0.0346	0.0252	0.0094	0.0142
H	0.0730	0.0177	0.0553	0.0301	0.0252	0.0075
H	0.0113	-0.0106	0.0219	0.0155	0.0064	0.0170
C	-0.0722	0.0043	-0.0765	-0.0901	0.0136	0.0093
H	0.0377	-0.0006	0.0383	0.0229	0.0154	0.0160
H	0.0670	0.0128	0.0542	0.0312	0.0230	0.0102
H	0.0464	0.0046	0.0418	0.0270	0.0148	0.0101
C	0.0261	0.0049	0.0212	0.0131	0.0082	0.0033
H	0.0560	0.0104	0.0455	0.0203	0.0252	0.0148
H	0.0596	0.0123	0.0473	0.0325	0.0148	0.0025
C	0.0262	0.0042	0.0220	0.0122	0.0098	0.0056
H	0.0564	0.0088	0.0476	0.0331	0.0146	0.0058
H	0.0539	0.0063	0.0476	0.0180	0.0296	0.0233
H	0.0963	-0.0071	0.1033	0.1057	-0.0023	0.0047
Cu	0.6720	0.4534	0.2186	0.0601	0.1585	-0.2949
Cl	-0.1496	0.2711	-0.4206	-0.4744	0.0079	-0.2173
Cation	N_{Ω}^{-}	0.0818		N_{Ω}^{+}	0.2714	
Anion	N_{Ω}^{-}	0.9182		N_{Ω}^{+}	0.7022	

Table S3. Electrophilic (N_{Ω}^{+}) and nucleophilic (N_{Ω}^{-}) Fukui function and dual description values for the IL [C_4mim][$CuAlCl_5$] (eV)

Atom	q_{N-1}	f^-	q_N	q_{N+1}	f^+	Δf
C	-0.0448	0.0006	-0.0454	-0.0526	0.0072	0.0066
C	0.0247	0.0032	0.0215	-0.0001	0.0215	0.0183
C	-0.0080	0.0031	-0.0112	-0.0400	0.0288	0.0257
N	0.0290	0.0057	0.0233	-0.0295	0.0528	0.0471
C	0.0120	0.0105	0.0015	-0.0614	0.0629	0.0524
C	0.0045	-0.0086	0.0131	-0.0374	0.0505	0.0591
N	0.0239	-0.0109	0.0348	-0.0144	0.0492	0.0601
C	0.0916	0.0009	0.0907	-0.0626	0.1533	0.1524
H	0.0101	-0.0072	0.0172	0.0128	0.0044	0.0116
H	0.0398	0.0071	0.0327	-0.0127	0.0454	0.0383
H	0.0618	0.0129	0.0489	-0.0293	0.0782	0.0653
H	0.0500	0.0021	0.0479	0.0256	0.0223	0.0202
H	0.0368	-0.0076	0.0444	0.0262	0.0182	0.0258
H	0.0720	0.0185	0.0535	-0.0128	0.0664	0.0479
H	0.0538	0.0025	0.0513	0.0286	0.0227	0.0202
H	0.0945	0.0119	0.0826	0.0390	0.0436	0.0316
H	0.0812	0.0032	0.0780	0.0481	0.0299	0.0267
H	0.0824	0.0060	0.0764	-0.0103	0.0867	0.0807
C	-0.0375	0.0013	-0.0388	-0.0454	0.0066	0.0052
H	0.0379	0.0093	0.0286	0.0153	0.0132	0.0039
H	0.0340	-0.0004	0.0344	0.0245	0.0099	0.0103
C	-0.0783	0.0025	-0.0808	-0.0874	0.0066	0.0041
H	0.0462	0.0090	0.0373	0.0220	0.0153	0.0063
H	0.0276	-0.0034	0.0311	0.0263	0.0048	0.0082
H	0.0355	0.0063	0.0292	0.0233	0.0058	-0.0005

Cl	-0.0785	0.0803	-0.1589	-0.1845	0.0256	-0.0547
Cl	-0.2115	0.0304	-0.2419	-0.2452	0.0033	-0.0271
Cl	-0.1904	0.0318	-0.2222	-0.2168	-0.0054	-0.0372
Cl	-0.1835	0.0521	-0.2355	-0.2590	0.0235	-0.0286
Al	0.3746	0.0073	0.3673	0.3596	0.0077	0.0004
Cu	0.7527	0.5408	0.2119	0.1996	0.0123	-0.5285
Cl	-0.2442	0.1782	-0.4224	-0.4372	0.0148	-0.1635
Cation	N_Ω^-	0.0787		N_Ω^+	0.2734	
Anion	N_Ω^-	0.9209		N_Ω^+	0.7194	

Table S4. Electrophilic (N_Ω^+) and nucleophilic (N_Ω^-) Fukui function and dual description values for the IL $[C_4mim][AlCl_4]$ (eV)

**Atom**	q_{N-1}	f^-	q_N	q_{N+1}	f^+	Δf
C	-0.0358	0.0008	-0.0366	-0.0402	0.0036	0.0028
C	0.0411	-0.0001	0.0412	0.0296	0.0116	0.0117
C	0.0213	0.0035	0.0178	-0.0078	0.0256	0.0221
N	0.0072	0.0020	0.0052	-0.0764	0.0817	0.0797
C	0.0296	0.0113	0.0182	-0.0325	0.0507	0.0394
C	0.0273	0.0116	0.0157	-0.0524	0.0681	0.0565
N	0.0075	0.0010	0.0065	-0.0824	0.0889	0.0878
C	0.1267	-0.0181	0.1448	-0.0491	0.1939	0.2120
H	0.0130	-0.0072	0.0202	0.0173	0.0029	0.0101
H	0.0339	0.0139	0.0200	0.0073	0.0127	-0.0011
H	0.0574	0.0142	0.0431	0.0198	0.0234	0.0091
H	0.0264	-0.0106	0.0370	0.0134	0.0236	0.0342
H	0.0453	-0.0058	0.0510	0.0171	0.0339	0.0397
H	0.0688	0.0194	0.0494	0.0126	0.0367	0.0173
H	0.0560	0.0029	0.0531	0.0237	0.0294	0.0264
H	0.0918	0.0127	0.0790	0.0353	0.0437	0.0309
H	0.0922	0.0128	0.0793	0.0272	0.0521	0.0392
H	0.0770	-0.0077	0.0847	0.0123	0.0724	0.0800
C	-0.0238	0.0011	-0.0249	-0.0317	0.0069	0.0058
H	0.0297	0.0111	0.0186	0.0071	0.0115	0.0003
H	0.0206	-0.0064	0.0270	0.0200	0.0071	0.0135
C	-0.0579	0.0040	-0.0619	-0.0689	0.0070	0.0030
H	0.0395	0.0104	0.0291	0.0131	0.0160	0.0056
H	0.0261	-0.0024	0.0285	0.0245	0.0040	0.0064
H	0.0301	0.0097	0.0204	0.0133	0.0071	-0.0026
Cl	-0.2244	0.0685	-0.2929	-0.3274	0.0344	-0.0341
Cl	-0.0360	0.2697	-0.3057	-0.3204	0.0147	-0.2550
Cl	-0.0360	0.2861	-0.3221	-0.3314	0.0092	-0.2769
Cl	-0.0263	0.2582	-0.2846	-0.3008	0.0162	-0.2420
Al	0.4723	0.0331	0.4393	0.4308	0.0084	-0.0246
Cation	N_Ω^-	0.0843		N_Ω^+	0.9144	
Anion	N_Ω^-	0.9156		N_Ω^+	0.0829	

Table S5. Electrophilic (N_Ω^+) and nucleophilic (N_Ω^-) Fukui function and dual description values for the IL $[(C_2H_5)_3NCu][AlCl_4]$ (eV)

**Atom**	q_{N-1}	f^-	q_N	q_{N+1}	f^+	Δf

C	-0.0534	0.0148	-0.0682	-0.0747	0.0064	-0.0084
C	0.0214	-0.0047	0.0261	0.0237	0.0024	0.0070
N	-0.0898	-0.0162	-0.0736	-0.0709	-0.0027	0.0135
C	-0.0555	0.0049	-0.0604	-0.0660	0.0056	0.0007
C	-0.0530	0.0060	-0.0591	-0.0684	0.0093	0.0033
C	0.0250	-0.0011	0.0260	0.0224	0.0037	0.0048
C	0.0246	-0.0014	0.0260	0.0218	0.0042	0.0057
Cu	0.9118	0.6373	0.2745	0.1490	0.1255	-0.5117
H	0.0587	0.0206	0.0381	0.0281	0.0101	-0.0105
H	0.0412	0.0128	0.0285	0.0215	0.0070	-0.0058
H	0.0410	0.0129	0.0281	0.0212	0.0069	-0.0060
H	0.0376	0.0024	0.0352	0.0321	0.0031	0.0007
H	0.0388	0.0033	0.0355	0.0316	0.0039	0.0006
H	0.0454	0.0152	0.0302	0.0207	0.0095	-0.0058
H	0.0681	0.0257	0.0425	0.0305	0.0119	-0.0137
H	0.0146	-0.0146	0.0292	0.0321	-0.0029	0.0117
H	0.0163	-0.0151	0.0314	0.0256	0.0058	0.0209
H	0.0682	0.0264	0.0418	0.0279	0.0139	-0.0125
H	0.0491	0.0165	0.0327	0.0219	0.0108	-0.0057
H	0.0432	0.0066	0.0365	0.0310	0.0056	-0.0011
H	0.0525	0.0275	0.0250	0.0111	0.0139	-0.0136
H	0.0538	0.0280	0.0257	0.0115	0.0143	-0.0138
H	0.0433	0.0070	0.0363	0.0287	0.0076	0.0005
Al	0.5130	0.0052	0.5078	0.3099	0.1980	0.1928
Cl	-0.2221	0.0411	-0.2632	-0.3824	0.1191	0.0780
Cl	-0.2097	0.0454	-0.2551	-0.3807	0.1256	0.0802
Cl	-0.2410	0.0470	-0.2880	-0.4287	0.1407	0.0936
Cl	-0.2430	0.0464	-0.2895	-0.4304	0.0064	-0.0084
Cation	N_{Ω}^{-}	0.8148		N_{Ω}^{+}	0.2757	
Anion	N_{Ω}^{-}	0.1852		N_{Ω}^{+}	0.7243	

Table S6. Electrophilic (N_{Ω}^{+}) and nucleophilic (N_{Ω}^{-}) Fukui function and dual description values for the IL benzene−$CuAlCl_4$ (eV)

**Atom**	q_{N-1}	f^-	q_N	q_{N+1}	f^+	Δf
Al	0.5084	0.0061	0.5023	0.4884	0.0138	0.0077
Cl	-0.2604	0.0240	-0.2844	-0.3075	0.0231	-0.0009
Cl	-0.2448	0.0030	-0.2478	-0.2516	0.0038	0.0008
Cl	-0.2333	0.0185	-0.2518	-0.2685	0.0167	-0.1531
Cl	-0.2590	0.0243	-0.2834	-0.3063	0.0229	-0.0014
Cu	0.3507	0.0330	0.3177	0.2319	0.0858	0.0528
C	0.0928	0.1173	-0.0245	-0.0496	0.0250	-0.0922
C	0.0646	0.0795	-0.0149	-0.1142	0.0993	0.0198
C	0.0480	0.0614	-0.0134	-0.1255	0.1121	0.0508
C	0.1844	0.1936	-0.0093	-0.0498	0.0406	-0.0018
C	0.0994	0.1121	-0.0128	-0.1197	0.1069	-0.0052
C	0.0236	0.0353	-0.0117	-0.1319	0.1203	0.0850
H	0.1075	0.0502	0.0573	0.0218	0.0355	-0.0148
H	0.1023	0.0456	0.0567	-0.0049	0.0616	0.0160
H	0.0976	0.0430	0.0547	-0.0069	0.0616	0.0186

H	0.1194	0.0643	0.0552	0.0162	0.0389	-0.0254
H	0.1053	0.0506	0.0547	-0.0045	0.0591	0.0085
H	0.0937	0.0378	0.0559	-0.0102	0.0661	0.0283
Cation	N_{Ω}^{-}	0.1090		N_{Ω}^{+}	0.1661	
Anion	N_{Ω}^{-}	0.9238		N_{Ω}^{-}	0.9128	

Table S7. Electrophilic (N_{Ω}^{+}) and nucleophilic (N_{Ω}^{-}) Fukui function and dual description values for the IL [C_4mimAg][$AlCl_4$] (eV)

**Atom**	q_{N-1}	f^-	q_N	q_{N+1}	f^+	Δf
C	-0.0392	0.0003	-0.0395	-0.0405	0.0010	0.0007
C	0.0369	0.0018	0.0351	0.0303	0.0047	0.0029
C	0.0253	0.0119	0.0134	-0.0314	0.0448	0.0329
N	-0.0023	0.0262	-0.0285	-0.0366	0.0082	-0.0181
C	0.0360	0.0387	-0.0027	-0.0290	0.0263	-0.0124
C	0.0291	0.0411	-0.0119	-0.0320	0.0201	-0.0210
N	-0.0056	0.0248	-0.0304	-0.0332	0.0028	-0.0220
C	0.0335	0.0421	-0.0086	-0.0201	0.0115	-0.0306
H	-0.0011	-0.0116	0.0105	0.0123	-0.0018	0.0098
H	0.0292	0.0107	0.0185	0.0132	0.0053	-0.0054
H	0.0578	0.0202	0.0376	0.0261	0.0115	-0.0087
H	0.0343	-0.0004	0.0347	0.0241	0.0106	0.0110
H	0.0675	0.0227	0.0448	-0.0388	0.0836	0.0609
H	0.0667	0.0210	0.0457	0.0021	0.0437	0.0227
H	0.0472	-0.0008	0.0480	-0.0201	0.0680	0.0689
H	0.0968	0.0264	0.0704	0.0538	0.0166	-0.0098
H	0.0960	0.0274	0.0686	0.0502	0.0184	-0.0090
C	-0.0269	0.0042	-0.0311	-0.0329	0.0018	-0.0023
H	0.0274	0.0130	0.0144	0.0088	0.0056	-0.0073
H	0.0198	0.0020	0.0178	0.0174	0.0005	-0.0016
C	-0.0591	0.0050	-0.0642	-0.0677	0.0035	-0.0015
H	0.0391	0.0135	0.0256	0.0173	0.0082	-0.0053
H	0.0183	-0.0025	0.0209	0.0204	0.0004	0.0030
H	0.0285	0.0077	0.0208	0.0156	0.0052	-0.0025
Cl	-0.2229	0.0456	-0.2685	-0.2903	0.0218	-0.0238
Cl	-0.1641	0.0655	-0.2296	-0.2705	0.0409	-0.0246
Cl	-0.2117	0.0400	-0.2517	-0.2629	0.0112	-0.0288
Cl	-0.2256	0.0427	-0.2684	-0.2824	0.0140	-0.0287
Al	0.4615	0.0022	0.4593	0.4304	0.0290	0.0268
Ag	0.7079	0.4587	0.2492	-0.1662	0.4154	-0.0433
Cation	N_{Ω}^{-}	0.8040		N_{Ω}^{+}	0.8161	
Anion	N_{Ω}^{-}	0.1960		N_{Ω}^{+}	0.1169	

Table S8. Electrophilic (N_{Ω}^{+}) and nucleophilic (N_{Ω}^{-}) Fukui function and dual description values for the IL ether−$CuAlCl_4$ (eV)

**Atom**	q_{N-1}	f^-	q_N	q_{N+1}	f^+	Δf
Al	0.5133	0.0094	0.5040	0.4400	0.0640	0.0547
Cl	-0.1968	0.0687	-0.2656	-0.2813	0.0157	-0.0530
Cl	-0.2387	0.0500	-0.2886	-0.3066	0.0179	-0.0321

Cl	-0.1968	0.0687	-0.2656	-0.2813	0.0157	-0.0530
C	-0.0533	0.0026	-0.0559	-0.0757	0.0198	0.0173
C	0.0873	0.0089	0.0784	0.0621	0.0164	0.0075
O	-0.2010	-0.0169	-0.1841	-0.1864	0.0023	0.0192
C	0.0873	0.0089	0.0784	0.0621	0.0164	0.0075
C	-0.0533	0.0026	-0.0559	-0.0757	0.0198	0.0173
Cu	0.9268	0.5975	0.3293	0.1752	0.1541	-0.4434
Cl	-0.2386	0.0500	-0.2886	-0.3066	0.0179	-0.0321
H	0.0800	0.0324	0.0476	-0.0026	0.0502	0.0178
H	0.0405	-0.0002	0.0407	0.0013	0.0394	0.0396
H	0.0368	-0.0032	0.0400	0.0087	0.0313	0.0345
H	0.0621	0.0227	0.0394	-0.0136	0.0530	0.0303
H	0.0625	0.0232	0.0393	-0.0116	0.0509	0.0277
H	0.0625	0.0232	0.0393	-0.0116	0.0509	0.0277
H	0.0621	0.0227	0.0394	-0.0136	0.0530	0.0303
H	0.0800	0.0324	0.0476	-0.0026	0.0502	0.0178
H	0.0368	-0.0032	0.0400	0.0088	0.0313	0.0345
H	0.0405	-0.0002	0.0407	0.0012	0.0395	0.0397
Cation	N_{Ω}^{-}	0.1556		N_{Ω}^{+}	0.5244	
Anion	N_{Ω}^{-}	0.8443		N_{Ω}^{+}	0.5854	

Table S9. Electrophilic (N_{Ω}^{+}) and nucleophilic (N_{Ω}^{-}) Fukui function and dual description values for the IL $(C_2H_5)_3NH][AgAlCl_5]$ (eV)

Atom	q_{N-1}	f^-	q_N	q_{N+1}	f^+	Δf
C	-0.0563	0.0024	-0.0588	-0.0612	0.0024	0.0000
C	0.0336	-0.0006	0.0342	0.0347	-0.0005	0.0001
N	0.0596	-0.0018	0.0614	0.0628	-0.0013	0.0005
H	0.0523	-0.0017	0.0540	0.0510	0.0029	0.0046
H	0.0459	0.0184	0.0275	0.0195	0.0079	-0.0105
H	0.0473	0.0054	0.0419	0.0398	0.0022	-0.0032
H	0.0095	-0.0182	0.0277	0.0330	-0.0053	0.0129
Al	0.4718	0.0036	0.4682	0.4084	0.0598	0.0562
Cl	-0.2347	0.0317	-0.2664	-0.2962	0.0298	-0.0019
Cl	-0.2341	0.0061	-0.2402	-0.2568	0.0166	0.0105
Cl	-0.2476	0.0166	-0.2641	-0.2850	0.0208	0.0043
Cl	-0.1743	0.0250	-0.1993	-0.2771	0.0779	0.0529
H	0.0374	0.0023	0.0350	0.0336	0.0014	-0.0009
C	-0.0560	0.0035	-0.0596	-0.0619	0.0024	-0.0012
H	0.0377	0.0019	0.0358	0.0328	0.0031	0.0012
H	0.0588	0.0126	0.0462	0.0385	0.0076	-0.0050
H	0.0328	0.0008	0.0319	0.0329	-0.0010	-0.0018
C	-0.0609	0.0023	-0.0632	-0.0705	0.0072	0.0049
H	0.0174	-0.0024	0.0199	0.0180	0.0019	0.0043
H	0.0644	0.0165	0.0479	0.0293	0.0186	0.0021
H	0.0176	-0.0146	0.0322	0.0290	0.0032	0.0178
C	0.0492	0.0044	0.0448	0.0425	0.0023	-0.0022
H	0.0524	0.0081	0.0443	0.0417	0.0026	-0.0055
H	0.0499	0.0067	0.0432	0.0379	0.0052	-0.0015
C	0.0467	0.0043	0.0424	0.0393	0.0031	-0.0012

H	0.0518	0.0090	0.0428	0.0354	0.0075	-0.0015
H	0.0545	0.0127	0.0418	0.0369	0.0049	-0.0078
H	0.1026	-0.0038	0.1064	0.1099	-0.0035	0.0003
Cl	0.1406	0.6152	-0.4746	-0.5440	0.0693	0.3712
Ag	0.5304	0.2334	0.2970	-0.3077	0.6046	-0.5459
Cation	N_Ω^-	0.0683		N_Ω^+	0.0748	
Anion	N_Ω^-	0.9316		N_Ω^+	0.8788	

Table S10. Electrophilic (N_Ω^+) and nucleophilic (N_Ω^-) Fukui function and dual description values for the IL [C_4mim][$CuCl_2$] (eV)

Atom	q_{N-1}	f^-	q_N	q_{N+1}	f^+	Δf
C	-0.0327	0.0022	-0.0349	-0.0424	0.0075	0.0053
C	0.0439	0.0015	0.0424	0.0302	0.0122	0.0108
C	0.0256	0.0066	0.0190	-0.0070	0.0260	0.0194
N	0.0061	0.0011	0.0050	-0.0765	0.0815	0.0804
C	0.0288	0.0133	0.0155	-0.0334	0.0489	0.0356
C	0.0285	0.0192	0.0093	-0.0613	0.0706	0.0514
N	0.0102	0.0064	0.0038	-0.0855	0.0893	0.0829
C	0.1354	-0.0212	0.1566	-0.0518	0.2084	0.2296
H	0.0411	0.0025	0.0386	0.0157	0.0229	0.0204
H	0.0342	0.0186	0.0156	0.0018	0.0138	-0.0049
H	0.0589	0.0149	0.0440	0.0141	0.0299	0.0150
H	0.0243	-0.0159	0.0402	0.0268	0.0134	0.0293
H	0.0622	0.0130	0.0493	0.0030	0.0463	0.0333
H	0.0628	0.0113	0.0515	0.0138	0.0376	0.0263
H	0.0498	-0.0027	0.0526	0.0350	0.0175	0.0202
H	0.0902	0.0116	0.0786	0.0357	0.0429	0.0313
H	0.0915	0.0146	0.0769	0.0228	0.0541	0.0395
H	0.0508	-0.0197	0.0706	0.0071	0.0634	0.0832
C	-0.0362	0.0009	-0.0371	-0.0349	-0.0022	-0.0030
H	0.0141	0.0117	0.0025	0.0052	-0.0028	-0.0144
H	-0.0138	-0.0261	0.0123	0.0146	-0.0022	0.0239
C	-0.0586	0.0041	-0.0627	-0.0725	0.0098	0.0056
H	0.0338	0.0074	0.0264	0.0142	0.0123	0.0049
H	0.0355	0.0173	0.0182	0.0060	0.0122	-0.0051
H	0.0304	-0.0004	0.0308	0.0190	0.0118	0.0121
Cu	0.8196	0.6132	0.2064	0.1866	0.0198	-0.5933
Cl	-0.3164	0.1538	-0.4702	-0.4936	0.0235	-0.1303
Cl	-0.3198	0.1411	-0.4609	-0.4901	0.0292	-0.1119
Cation	N_Ω^-	0.0919		N_Ω^+	0.9249	
Anion	N_Ω^-	0.9081		N_Ω^+	0.0725	

Table S11. Electrophilic (N_Ω^+) and nucleophilic (N_Ω^-) Fukui function and dual description values for the IL [$(C_2H_5)_3NH$][$ZnCl_3$] (eV)

Atom	q_{N-1}	f^-	q_N	q_{N+1}	f^+	Δf
C	-0.0572	0.0045	-0.0617	-0.1198	0.0582	0.0537
C	0.0396	0.0010	0.0386	0.0302	0.0085	0.0075
N	0.0581	-0.0019	0.0600	0.0590	0.0010	0.0030

H	0.0542	0.0075	0.0467	-0.0039	0.0506	0.0431
H	0.0374	0.0053	0.0321	-0.0661	0.0983	0.0930
H	0.0443	0.0000	0.0443	0.0300	0.0143	0.0143
H	0.0445	0.0002	0.0443	0.0301	0.0142	0.0140
H	0.0374	0.0053	0.0320	-0.0664	0.0984	0.0931
C	-0.0576	0.0015	-0.0591	-0.0769	0.0178	0.0163
H	0.0390	0.0076	0.0314	0.0118	0.0196	0.0120
H	0.0528	0.0067	0.0461	0.0091	0.0371	0.0304
H	0.0265	-0.0095	0.0359	0.0201	0.0158	0.0253
C	-0.0576	0.0016	-0.0591	-0.0768	0.0177	0.0161
H	0.0275	-0.0089	0.0364	0.0205	0.0159	0.0248
H	0.0527	0.0067	0.0460	0.0095	0.0365	0.0297
H	0.0389	0.0076	0.0314	0.0121	0.0193	0.0117
C	0.0422	0.0019	0.0402	0.0096	0.0306	0.0287
H	0.0412	-0.0005	0.0418	0.0025	0.0393	0.0398
H	0.0483	0.0099	0.0384	-0.0869	0.1253	0.1155
C	0.0424	0.0019	0.0405	0.0105	0.0300	0.0281
H	0.0483	0.0098	0.0386	-0.0855	0.1241	0.1143
H	0.0412	-0.0006	0.0418	0.0040	0.0379	0.0385
H	0.1021	-0.0056	0.1077	0.1011	0.0066	0.0122
Zn	0.5103	0.0720	0.4383	0.4117	0.0266	-0.0454
Cl	-0.3135	0.0600	-0.3735	-0.3810	0.0075	-0.0525
Cl	-0.3087	0.0586	-0.3673	-0.3760	0.0087	-0.0499
Cl	0.3661	0.7576	-0.3915	-0.4141	0.0226	-0.7351
Cation	N_Ω^-	0.0518		N_Ω^+	0.9168	
Anion	N_Ω^-	0.9481		N_Ω^+	0.0653	

Table S12. Electrophilic (N_Ω^+) and nucleophilic (N_Ω^-) Fukui function and dual description values for the IL $[(C_2H_5)_3NH][ZnAlCl_6]$ (eV)

Atom	q_{N-1}	f^-	q_N	q_{N+1}	f^+	Δf
C	-0.0726	0.0049	-0.0775	-0.1084	0.0309	0.0260
C	0.0191	-0.0003	0.0193	0.0126	0.0067	0.0070
N	0.0715	-0.0004	0.0719	0.0718	0.0001	0.0004
H	0.0632	0.0071	0.0561	0.0271	0.0290	0.0219
H	0.0476	0.0078	0.0398	-0.0152	0.0550	0.0472
H	0.0521	0.0013	0.0508	0.0378	0.0130	0.0117
H	0.0433	-0.0046	0.0479	0.0372	0.0107	0.0153
Al	0.3928	0.0086	0.3842	0.2980	0.0862	0.0813
Cl	-0.1981	0.0288	-0.2269	-0.2607	0.0338	0.0050
Cl	-0.1336	0.0213	-0.1549	-0.1823	0.0275	0.0062
Cl	-0.1885	0.0385	-0.2269	-0.2761	0.0492	0.0107
Cl	-0.1094	0.0285	-0.1379	-0.1768	0.0389	0.0105
H	0.0440	0.0047	0.0393	-0.0152	0.0545	0.0498
C	-0.0749	0.0028	-0.0778	-0.0942	0.0164	0.0136
H	0.0438	0.0059	0.0379	0.0157	0.0221	0.0162
H	0.0645	0.0097	0.0549	0.0235	0.0314	0.0217
H	0.0374	-0.0046	0.0421	0.0294	0.0127	0.0173
C	-0.0787	0.0008	-0.0795	-0.0961	0.0166	0.0158
H	0.0187	-0.0141	0.0329	0.0214	0.0115	0.0257

H	0.0594	0.0049	0.0544	0.0223	0.0321	0.0272
H	0.0457	0.0079	0.0378	0.0152	0.0227	0.0148
C	0.0205	0.0024	0.0181	-0.0075	0.0256	0.0232
H	0.0441	0.0024	0.0417	-0.0015	0.0432	0.0407
H	0.0530	0.0090	0.0440	-0.0380	0.0820	0.0730
C	0.0234	0.0018	0.0216	-0.0052	0.0269	0.0251
H	0.0571	0.0109	0.0462	-0.0461	0.0923	0.0777
H	0.0412	0.0003	0.0409	0.0007	0.0402	0.0399
H	0.1028	-0.0034	0.1062	0.1033	0.0029	0.0063
Cl	-0.1788	0.1309	-0.3098	-0.3272	0.0174	-0.1135
Zn	0.4272	0.0664	0.3608	0.3371	0.0237	-0.5840
Cl	0.2625	0.6198	-0.3573	-0.3932	0.0359	-0.0427
Cation	N_Ω^-	0.0572		N_Ω^+	0.6783	
Anion	N_Ω^-	0.9427		N_Ω^+	0.3126	

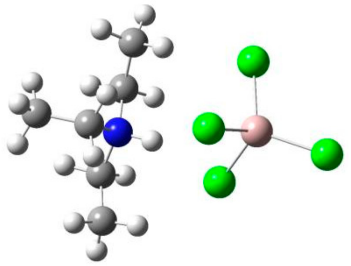

Figure S1. Structure of [$(C_2H_5)_3NH$][$AlCl_4$]

Table S13. Cartesian coordinates of optimized [$(C_2H_5)_3NH$][$AlCl_4$]

**[$(C_2H_5)_3NH$][$AlCl_4$]**			
C	-3.6927	-1.3407	-0.5554
C	-2.1765	-1.2346	-0.6636
N	-1.7341	0.1625	-0.5709
H	-3.9469	-2.4053	-0.6484
H	-4.1701	-0.8295	-1.4043
H	-1.7933	-1.6334	-1.6084
H	-1.6750	-1.7697	0.1464
Al	3.9517	-1.6783	-1.2876
Cl	2.0602	-0.8452	-2.0122
Cl	3.5044	-3.6718	-0.5153

Cl	4.5543	-0.4891	0.4550
Cl	5.4557	-1.6984	-2.8022
H	-4.0759	-0.9546	0.3660
C	-2.8212	0.8687	-2.7682
H	-3.8658	0.8062	-2.5448
H	-2.6530	1.6600	-3.4686
H	-2.4907	-0.0573	-3.1902
C	-0.4268	-0.0776	1.6170
H	0.1775	-0.8806	1.2498
H	0.1770	0.5848	2.2015
H	-1.2139	-0.4726	2.2248
C	-2.0356	1.1527	-1.4745
H	-1.0695	1.5460	-1.7127
H	-2.5800	1.8697	-0.8961
C	-1.0328	0.6934	0.4296
H	-1.7230	1.4227	0.7993
H	-0.2534	1.1838	-0.1153
H	-0.4608	-0.0438	-1.1977

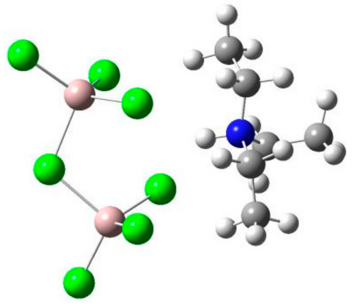

Figure S2. Structure of [$(C_2H_5)_3NH$][Al_2Cl_7]

Table S14. Cartesian coordinates of optimized [$(C_2H_5)_3NH$][Al_2Cl_7]

**[$(C_2H_5)_3NH$][Al_2Cl_7]**			
C	4.5779	-1.4208	-1.2383
C	3.0973	-1.0461	-1.1537
N	2.6399	-0.8357	0.2841
H	4.8079	-1.6957	-2.2716
H	4.8200	-2.2759	-0.5996
H	2.4477	-1.8194	-1.5671
H	2.8764	-0.1147	-1.6793
Al	-0.4446	2.1042	-0.0774
Cl	0.7953	1.6176	-1.8134
Cl	-0.7381	4.2121	0.2823
Cl	0.3240	0.9754	1.6614
H	5.2224	-0.5803	-0.9657
C	2.2881	-3.4088	0.3674
H	3.0932	-3.6719	-0.3231
H	2.2006	-4.2135	1.1046
H	1.3433	-3.3415	-0.1718
C	3.6020	1.5482	0.3385
H	2.6331	2.0394	0.2514

H	4.2484	2.1849	0.9504
H	4.0461	1.4653	-0.6569
C	2.5921	-2.1193	1.1365
H	1.8254	-1.9231	1.8925
H	3.5668	-2.1921	1.6265
C	3.4808	0.1958	1.0407
H	4.4613	-0.2661	1.1770
H	2.9954	0.3116	2.0129
Cl	-2.5111	1.1283	-0.4221
Al	-2.3876	-1.1951	-0.0426
Cl	-0.3310	-1.5845	-0.7634
Cl	-3.9321	-2.0338	-1.3003
Cl	-2.5327	-1.4851	2.0974
H	1.6603	-0.4800	0.2317

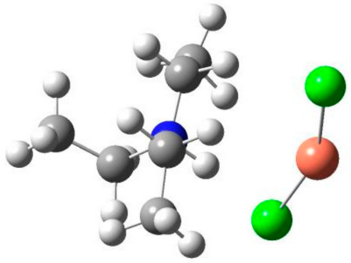

Figure S3. Structure of [$(C_2H_5)_3NH$][$CuCl_2$]

Table S15. Cartesian coordinates of optimized [$(C_2H_5)_3NH$][$CuCl_2$]

**[$(C_2H_5)_3NH$][$CuCl_2$]**			
C	-3.8343	2.7188	0.9335
C	-2.6737	1.7279	0.8589
N	-2.8835	0.6664	-0.2186
H	-3.6362	3.4117	1.7560
H	-4.7874	2.2212	1.1376
H	-2.5373	1.1860	1.7961
H	-1.7257	2.2193	0.6277
H	-3.9228	3.3099	0.0177
C	-3.8684	-0.9855	1.4264
H	-4.0584	-0.3035	2.2588
H	-4.5889	-1.8055	1.4890
H	-2.8619	-1.4048	1.5282
C	-1.8011	2.0876	-1.9990
H	-0.8779	1.5253	-1.8296
H	-1.8535	2.3255	-3.0650
H	-1.7448	3.0226	-1.4367
C	-4.0304	-0.2945	0.0692
H	-3.9984	-1.0286	-0.7415
H	-4.9560	0.2815	0.0025
C	-3.0197	1.2448	-1.6227
H	-3.9488	1.8180	-1.6417
H	-3.1059	0.3802	-2.2857
H	-2.0193	0.0891	-0.2128

Cu	0.0469	-1.8787	-0.4165
Cl	1.4366	-0.2419	-0.6513
Cl	-1.3427	-3.5155	-0.1818

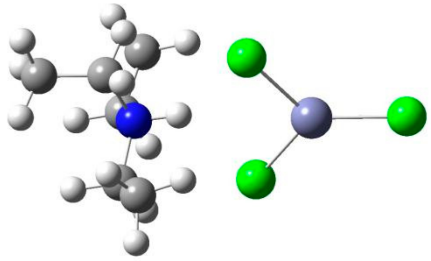

Figure S4. Structure of [$(C_2H_5)_3NH$][$ZnCl_3$]

Table S16. Cartesian coordinates of optimized [$(C_2H_5)_3NH$][$ZnCl_3$]

**[$(C_2H_5)_3NH$][$ZnCl_3$]**			
C	-3.8617	0.3884	-0.0014
C	-2.6597	-0.5380	-0.0009
N	-1.3512	0.1822	-0.0004
H	-4.7711	-0.2214	-0.0020
H	-3.8867	1.0249	-0.8931
H	-2.6492	-1.1893	-0.8791
H	-2.6498	-1.1892	0.8775
H	-3.8876	1.0245	0.8906
C	-1.1978	0.1881	-2.5048
H	-2.2336	-0.0669	-2.7565
H	-0.7892	0.7835	-3.3277
H	-0.6067	-0.7333	-2.4255
C	-1.2024	0.1917	2.5042
H	-0.6109	-0.7296	2.4279
H	-0.7959	0.7886	3.3272
H	-2.2388	-0.0631	2.7539
C	-1.0923	1.0026	-1.2275
H	-0.0780	1.4002	-1.1009
H	-1.7938	1.8443	-1.2107
C	-1.0940	1.0043	1.2260
H	-1.7950	1.8464	1.2066
H	-0.0793	1.4011	1.1005
H	-0.6398	-0.5905	0.0006
Zn	1.4156	-2.4109	-0.3557
Cl	1.6516	-0.3342	-1.1612
Cl	-0.4562	-3.5683	-0.7735
Cl	3.0514	-3.3303	0.8676

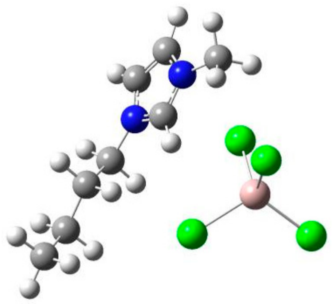

Figure S5. Structure of [C_4mim][$AlCl_4$]

Table S17. Cartesian coordinates of optimized [C_4mim][$AlCl_4$]

**[C_4mim][$AlCl_4$]**			
C	3.1051	0.5583	0.2375
C	2.4076	-0.1449	-0.9159
C	-0.7426	-3.1288	1.4467
N	1.6913	-1.3578	-0.4900
C	1.6374	-2.5316	-1.1996
C	0.7650	-3.3531	-0.5505
N	0.3053	-2.6613	0.5425
C	0.8611	-1.4494	0.5502
H	2.3764	0.7713	1.0291
H	3.8824	-0.0966	0.6564
H	3.1308	-0.4715	-1.6708
H	1.6690	0.5154	-1.3839
H	-1.7172	-2.9448	0.9840
H	-0.5852	-4.1946	1.6309
H	-0.6817	-2.5675	2.3800
H	2.2150	-2.6812	-2.1014
H	0.4360	-4.3593	-0.7718
H	0.6344	-0.6620	1.2603
C	3.7081	1.8809	-0.2295
H	4.4565	1.6898	-1.0123
H	2.9106	2.4838	-0.6846
C	4.3423	2.6549	0.9213
H	4.7671	3.6025	0.5716
H	3.5902	2.8826	1.6875
H	5.1464	2.0746	1.3929
Cl	-3.4707	2.0621	-0.6981
Cl	-2.1490	-0.1482	1.8242
Cl	-1.3942	-0.7265	-1.5656
Cl	0.0146	2.0011	0.1676
Al	-1.8250	0.8439	-0.0980

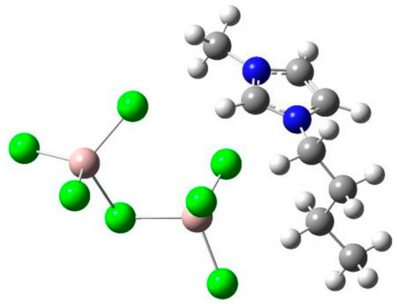

Figure S6. Structure of [C_4mim][Al_2Cl_7]

Table S18. Cartesian coordinates of optimized [C_4mim][Al_2Cl_7]

**[C_4mim][Al_2Cl_7]**			
C	-4.2394	0.5443	1.3465
C	-3.3317	-0.5705	1.8500
C	-0.5906	-3.6184	-0.9275
N	-2.9140	-1.4823	0.7671
C	-3.7301	-1.9736	-0.2251
C	-2.9861	-2.8527	-0.9529
N	-1.7363	-2.8895	-0.3852
C	-1.7063	-2.0363	0.6393
H	-4.5547	1.1116	2.2328
H	-5.1611	0.1189	0.9220
H	-3.8384	-1.1792	2.6079
H	-2.4113	-0.1603	2.2741
H	-0.9719	-4.4930	-1.4596
H	-0.0339	-2.9591	-1.6008
H	0.0554	-3.9325	-0.1058
H	-4.7605	-1.6675	-0.3333
H	-3.2381	-3.4450	-1.8219
H	-0.8318	-1.8019	1.2383
C	-3.5715	1.4852	0.3464
H	-3.1500	0.9100	-0.4908
H	-2.7304	1.9855	0.8417
C	-4.5399	2.5304	-0.1951
H	-4.0232	3.2064	-0.8846
H	-5.3705	2.0565	-0.7354
H	-4.9642	3.1309	0.6201
Al	-0.0145	1.3549	-0.5969
Cl	-0.8582	-0.1828	-1.8620
Cl	-0.1369	0.9157	1.5109
Cl	-0.7489	3.2881	-1.1119
Al	3.2554	-0.2083	0.3126
Cl	1.8536	-1.8599	0.3517
Cl	4.9996	-0.6393	-0.8266
Cl	3.5377	0.7348	2.1932
Cl	2.2107	1.3767	-1.0536

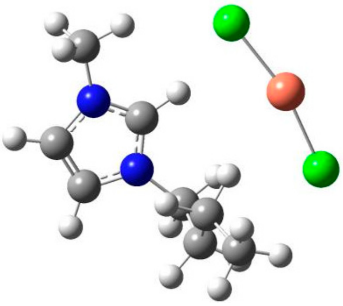

Figure S7. Structure of [C_4mim][$CuCl_2$]

Table S19. Cartesian coordinates of optimized [C_4mim][$CuCl_2$]

[C_4mim][$CuCl_2$]			
C	-4.2394	0.5443	1.3465
C	-3.3317	-0.5705	1.8500
C	-0.5906	-3.6184	-0.9275
N	-2.9140	-1.4823	0.7671
C	-3.7301	-1.9736	-0.2251
C	-2.9861	-2.8527	-0.9529
N	-1.7363	-2.8895	-0.3852
C	-1.7063	-2.0363	0.6393
H	-4.5547	1.1116	2.2328
H	-5.1611	0.1189	0.9220
H	-3.8384	-1.1792	2.6079
H	-2.4113	-0.1603	2.2741
H	-0.9719	-4.4930	-1.4596
H	-0.0339	-2.9591	-1.6008
H	0.0554	-3.9325	-0.1058
H	-4.7605	-1.6675	-0.3333
H	-3.2381	-3.4450	-1.8219
H	-0.8318	-1.8019	1.2383
C	-3.5715	1.4852	0.3464
H	-3.1500	0.9100	-0.4908
H	-2.7304	1.9855	0.8417
C	-4.5399	2.5304	-0.1951
H	-4.0232	3.2064	-0.8846
H	-5.3705	2.0565	-0.7354
H	-4.9642	3.1309	0.6201
Cu	1.5484	-0.7022	3.0638
Cl	0.2987	0.7701	4.0314
Cl	2.7982	-2.1744	2.0962

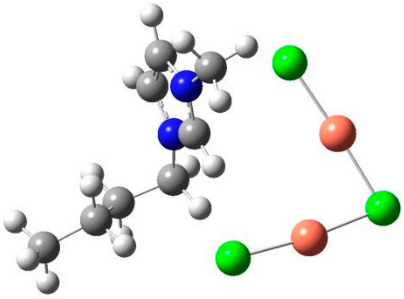

Figure S8. Structure of [C4mim][Cu2Cl3]

Table S20. Cartesian coordinates of optimized [C4mim][Cu2Cl3]

**[C4mim][Cu2Cl3]**			
C	-3.9804	0.5842	1.3465
C	-3.0726	-0.5306	1.8500
C	-0.3315	-3.5785	-0.9275
N	-2.6550	-1.4425	0.7671
C	-3.4711	-1.9337	-0.2251
C	-2.7270	-2.8128	-0.9529
N	-1.4773	-2.8496	-0.3852
C	-1.4472	-1.9965	0.6393
H	-4.2956	1.1514	2.2328
H	-4.9021	0.1588	0.9220
H	-3.5793	-1.1393	2.6079
H	-2.1523	-0.1204	2.2741
H	-0.7128	-4.4532	-1.4596
H	0.2252	-2.9193	-1.6008
H	0.3144	-3.8926	-0.1058
H	-4.5014	-1.6277	-0.3333
H	-2.9791	-3.4052	-1.8219
H	-0.5727	-1.7621	1.2383
C	-3.3125	1.5251	0.3464
H	-2.8910	0.9498	-0.4908
H	-2.4714	2.0254	0.8417
C	-4.2809	2.5703	-0.1951
H	-3.7641	3.2463	-0.8846
H	-5.1114	2.0964	-0.7354

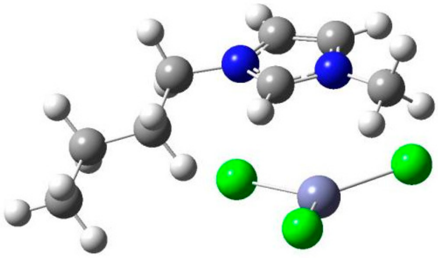

Figure S9. Structure of [C4mim][ZnCl3]

Table S21. Cartesian coordinates of optimized [C4mim][ZnCl3]

**[C4mim][ZnCl3]**			
C	-0.7420	0.9054	0.1514
N	-1.7808	1.6795	-0.1932
C	-1.3778	3.0042	-0.2056
C	-0.0585	3.0216	0.1419
N	0.3169	1.7068	0.3591
C	-3.1260	1.1710	-0.5045
C	1.6550	1.2257	0.7620
C	2.2540	0.2364	-0.2424

C	3.6527	-0.2264	0.1894
C	4.2640	-1.2307	-0.7948
H	-0.7165	-0.2155	0.2370
H	-2.0539	3.8069	-0.4574
H	0.6334	3.8425	0.2531
H	-3.3934	1.4680	-1.5224
H	-3.0939	0.0765	-0.4169
H	-3.8418	1.5946	0.2055
H	1.5577	0.7601	1.7480
H	2.2822	2.1164	0.8693
H	2.3067	0.7153	-1.2307
H	1.5882	-0.6314	-0.3254
H	3.5883	-0.6885	1.1846
H	4.3203	0.6428	0.2905
H	5.2586	-1.5523	-0.4642

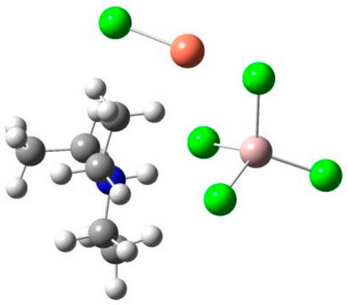

Figure S10. Structure of $[(C_2H_5)_3NH][CuAlCl_5]$

Table S22. Cartesian coordinates of optimized $[(C_2H_5)_3NH][CuAlCl_5]$

**$[(C_2H_5)_3NH][CuAlCl_5]$**			
C	-4.1216	-0.5418	-0.5931
C	-2.6111	-0.4604	-0.6885
N	-1.8982	-1.3422	0.3002
H	-4.5331	0.1127	-1.3684
H	-4.4960	-0.1741	0.3685
H	-2.2580	0.5620	-0.5201
H	-2.2502	-0.7622	-1.6740
Al	2.1034	-0.5586	-0.0465
Cl	1.1345	-1.8787	1.3951
Cl	0.6397	-0.3041	-1.6523
Cl	4.0207	-1.2146	-0.6569
Cl	2.1936	1.4659	0.8671
H	-4.5050	-1.5541	-0.7692
C	-1.7557	0.4099	2.0962
H	-2.3024	1.1812	1.5438
H	-1.9367	0.5560	3.1664
H	-0.6781	0.5407	1.9268
C	-1.7850	-3.2521	-1.3358
H	-0.7947	-2.8932	-1.6411
H	-1.7748	-4.3466	-1.3619
H	-2.5312	-2.9052	-2.0581

C	-2.1768	-1.0004	1.7339
H	-1.6127	-1.7301	2.3230
H	-3.2456	-1.1719	1.8970
C	-2.1064	-2.8103	0.0799
H	-3.1425	-3.0313	0.3577
H	-1.4408	-3.3043	0.7949
H	-0.8922	-1.1740	0.1493
Cu	0.2130	2.2239	-0.0756
Cl	-1.7827	3.0014	-0.5527

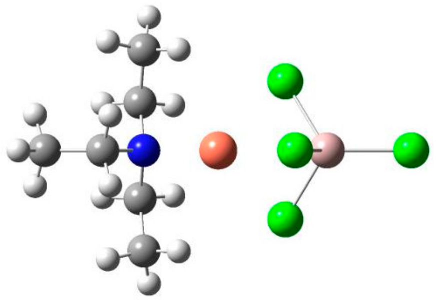

Figure S11. Structure of [$(C_2H_5)_3$NCu][$AlCl_4$]

Table S23. Cartesian coordinates of optimized [$(C_2H_5)_3$NCu][$AlCl_4$]

**[$(C_2H_5)_3$NCu][$AlCl_4$]**			
C	-4.2246	0.4676	0.8197
C	-2.7631	0.1652	1.0941
N	-1.9638	-0.0620	-0.1511
H	-4.7080	0.7259	1.7677
H	-4.7541	-0.3962	0.4026
H	-2.6415	-0.7338	1.7051
H	-2.2678	0.9793	1.6270
Al	1.8955	-0.0314	0.0607
Cl	0.8615	-1.4479	-1.2628
Cl	0.5609	0.0412	1.8262
Cl	1.8026	1.9002	-0.9045
Cl	3.8500	-0.6818	0.5948
H	-4.3466	1.3201	0.1409
C	-2.4012	-2.5253	-0.1906
H	-3.1851	-2.5799	0.5730
H	-2.5549	-3.3534	-0.8901
H	-1.4193	-2.6616	0.2792
C	-1.5337	2.4329	-0.2522
H	-0.7133	2.2985	0.4611
H	-1.2161	3.1966	-0.9696
H	-2.4255	2.7992	0.2693
C	-2.4403	-1.2221	-0.9680
H	-1.7678	-1.2658	-1.8283
H	-3.4471	-0.9743	-1.3196
C	-1.8072	1.1552	-1.0248
H	-2.7211	1.2299	-1.6251
H	-0.9682	0.9171	-1.6884

Cu	-0.2461	-0.4911	0.4507

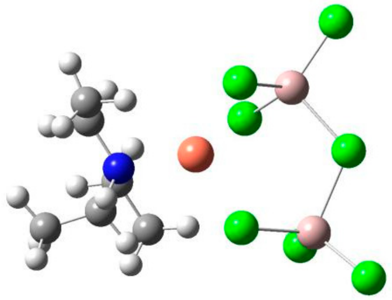

Figure S12. Structure of [$(C_2H_5)_3NCu$][Al_2Cl_7]

Table S24. Cartesian coordinates of optimized [$(C_2H_5)_3NCu$][Al_2Cl_7]

**[$(C_2H_5)_3NCu$][Al_2Cl_7]**			
C	-3.0139	-3.3517	-0.8740
C	-2.2880	-2.0019	-0.9421
N	-2.6962	-1.0219	0.1329
H	-2.7489	-3.9400	-1.7572
H	-4.1019	-3.2313	-0.8608
H	-2.4774	-1.5158	-1.9062
H	-1.2069	-2.1443	-0.8672
Al	0.2733	1.8210	0.1942
Cl	-1.4807	1.9616	1.7125
Cl	-0.1643	0.6640	-1.7425
Cl	1.6180	0.4206	1.3654
Cl	1.0945	3.7601	-0.1887
H	-2.7120	-3.9228	0.0087
C	-3.8522	0.7982	-1.0282
H	-3.3137	0.5445	-1.9468
H	-4.8079	1.2248	-1.3307
H	-3.3268	1.5871	-0.4638
C	-1.1865	-2.0749	1.8372
H	-0.4747	-1.2589	1.6763
H	-1.1314	-2.3691	2.8892
H	-0.8754	-2.9234	1.2220
C	-4.0628	-0.4412	-0.1412
H	-4.4952	-0.1376	0.8174
H	-4.7272	-1.1722	-0.6101
C	-2.6093	-1.6107	1.5123
H	-3.3238	-2.4375	1.6087
H	-2.9026	-0.8180	2.2099
Al	2.8764	-0.9857	-0.1435
Cl	4.4642	-1.7240	1.1218
Cl	3.3536	0.4970	-1.6552
Cl	1.3482	-2.4528	-0.6751
Cu	-1.7704	0.6122	-0.1177

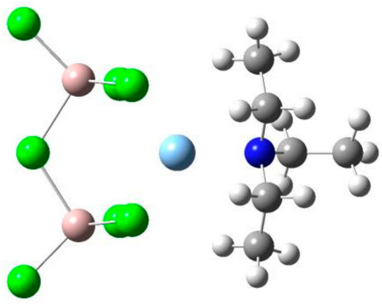

Figure S13. Structure of $[(C_2H_5)_3NAg][Al_2Cl_7]$

Table S25. Cartesian coordinates of optimized $[(C_2H_5)_3NAg][Al_2Cl_7]$

$[(C_2H_5)_3NAg][Al_2Cl_7]$			
C	4.5779	-1.4208	-1.2383
C	3.0973	-1.0461	-1.1537
N	2.6399	-0.8357	0.2841
H	4.8079	-1.6957	-2.2716
H	4.8200	-2.2759	-0.5996
H	2.4477	-1.8194	-1.5671
H	2.8764	-0.1147	-1.6793
Al	-1.0468	2.5232	0.2810
Cl	0.1931	2.0366	-1.4550
Cl	-1.3403	4.6311	0.6406
Cl	-0.2783	1.3944	2.0198
H	5.2224	-0.5803	-0.9657
C	2.2881	-3.4088	0.3674
H	3.0932	-3.6719	-0.3231
H	2.2006	-4.2135	1.1046
H	1.3433	-3.3415	-0.1718
C	3.6020	1.5482	0.3385
H	2.6331	2.0394	0.2514
H	4.2484	2.1849	0.9504
H	4.0461	1.4653	-0.6569
C	2.5921	-2.1193	1.1365
H	1.8254	-1.9231	1.8925
H	3.5668	-2.1921	1.6265
C	3.4808	0.1958	1.0407
H	4.4613	-0.2661	1.1770
H	2.9954	0.3116	2.0129
Cl	-3.1133	1.5473	-0.0637
Al	-2.9899	-0.7761	0.3158
Cl	-0.9332	-1.1655	-0.4050
Cl	-4.5343	-1.6149	-0.9419
Cl	-3.1349	-1.0661	2.4558
Ag	0.7248	-0.1403	0.1817

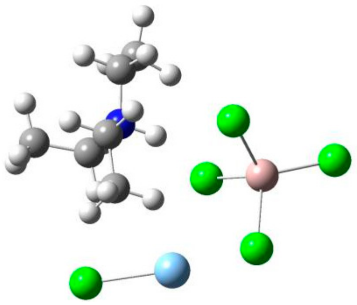

Figure S14. Structure of [$(C_2H_5)_3NH$][$AgAlCl_5$]

Table S26. Cartesian coordinates of optimized [$(C_2H_5)_3NH$][$AgAlCl_5$]

**[$(C_2H_5)_3NH$][$AgAlCl_5$]**			
C	-3.9325	1.3418	-0.5602
C	-2.4470	1.0684	-0.6847
N	-1.6078	1.8440	0.2936
H	-4.4361	0.7712	-1.3473
H	-4.1852	2.4006	-0.6957
H	-2.0658	1.3213	-1.6763
H	-2.2380	0.0072	-0.5200
Al	2.2474	0.4506	-0.0336
Cl	4.2143	0.8600	-0.7027
Cl	0.7420	0.3629	-1.6278
Cl	1.4670	1.9294	1.3718
Cl	2.1009	-1.5363	0.9298
H	-4.3337	0.9895	0.3962
C	-1.1684	3.6910	-1.3571
H	-1.9438	3.4637	-2.0958
H	-0.9785	4.7687	-1.3904
H	-0.2425	3.1723	-1.6348
C	-1.7506	0.1066	2.1006
H	-0.7091	-0.1971	1.9226
H	-1.9407	-0.0017	3.1737
H	-2.4239	-0.5678	1.5597
C	-1.5897	3.3229	0.0542
H	-0.8756	3.7229	0.7806
H	-2.5880	3.6991	0.3020
C	-1.9377	1.5644	1.7297
H	-2.9646	1.9085	1.8896
H	-1.2625	2.1971	2.3138
Cl	-0.6190	3.5070	-1.5223
Al	-2.3353	-1.0828	0.0079
Al	-0.5623	1.9867	-0.0211

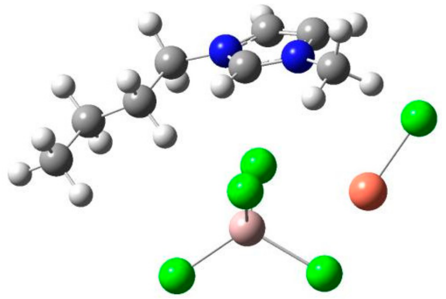

Figure S15. Structure of [C_4mim][$CuAlCl_5$]

Table S27. Cartesian coordinates of optimized [C_4mim][$CuAlCl_5$]

**[C_4mim][$CuAlCl_5$]**			
C	3.1225	-1.3714	0.0567
C	2.3400	-2.3259	-0.8316
C	-1.6686	-2.1920	2.0469
N	0.9936	-2.6104	-0.3096
C	-0.0485	-3.1207	-1.0529
C	-1.1725	-3.0326	-0.2873
N	-0.7914	-2.4734	0.9105
C	0.5158	-2.2218	0.8707
H	2.4943	-0.5038	0.2999
H	3.3909	-1.8638	1.0030
H	2.8669	-3.2792	-0.9569
H	2.1885	-1.8713	-1.8170
H	-2.6073	-1.7937	1.6502
H	-1.8423	-3.1136	2.6111
H	-1.1787	-1.4430	2.6741
H	0.0891	-3.4600	-2.0704
H	-2.2085	-3.2576	-0.5039
H	1.0754	-1.7381	1.6580
C	4.3857	-0.8778	-0.6438
H	5.0081	-1.7355	-0.9374
H	4.0925	-0.3587	-1.5671
C	5.1836	0.0723	0.2438
H	6.0802	0.4318	-0.2732
H	4.5747	0.9432	0.5170
H	5.5016	-0.4297	1.1670
Cl	-1.4440	2.8889	-0.0080
Cl	0.5079	0.8545	2.0719
Cl	0.1832	0.2304	-1.4255
Cl	2.1988	2.9479	-0.2614
Al	0.4654	1.7658	0.0991
Cu	-2.5250	0.8586	-0.3550
Cl	-3.7822	-0.9437	-0.4398

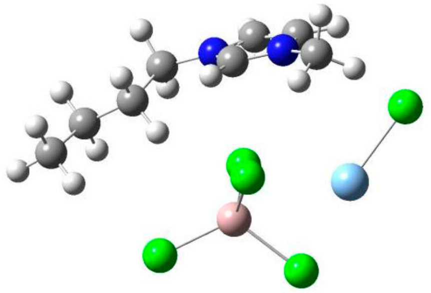

Figure S16. Structure of [C_4mim][$AgAlCl_5$]

Table S28. Cartesian coordinates of optimized [C_4mim][$AgAlCl_5$]

**[C_4mim][$AgAlCl_5$]**			
C	3.1225	-1.3714	0.0567
C	2.3400	-2.3259	-0.8316
C	-1.6686	-2.1920	2.0469
N	0.9936	-2.6104	-0.3096
C	-0.0485	-3.1207	-1.0529
C	-1.1725	-3.0326	-0.2873
N	-0.7914	-2.4734	0.9105
C	0.5158	-2.2218	0.8707
H	2.4943	-0.5038	0.2999
H	3.3909	-1.8638	1.0030
H	2.8669	-3.2792	-0.9569
H	2.1885	-1.8713	-1.8170
H	-2.6073	-1.7937	1.6502
H	-1.8423	-3.1136	2.6111
H	-1.1787	-1.4430	2.6741
H	0.0891	-3.4600	-2.0704
H	-2.2085	-3.2576	-0.5039
H	1.0754	-1.7381	1.6580
C	4.3857	-0.8778	-0.6438
H	5.0081	-1.7355	-0.9374
H	4.0925	-0.3587	-1.5671
C	5.1836	0.0723	0.2438
H	6.0802	0.4318	-0.2732
H	4.5747	0.9432	0.5170
H	5.5016	-0.4297	1.1670
Cl	-1.4440	2.8889	-0.0080
Cl	0.5079	0.8545	2.0719
Cl	0.1832	0.2304	-1.4255
Cl	2.1988	2.9479	-0.2614
Al	0.4654	1.7658	0.0991
Cl	-3.7822	-0.9437	-0.4398
Ag	-2.4501	0.9659	-0.3500
